# Anagen hair follicles transplanted into mature human scars remodel fibrotic tissue

**DOI:** 10.1038/s41536-022-00270-3

**Published:** 2023-01-06

**Authors:** Magdalena Plotczyk, Francisco Jiménez, Summik Limbu, Colin J. Boyle, Jesse Ovia, Benjamin D. Almquist, Claire A. Higgins

**Affiliations:** 1 Department of Bioengineering, Imperial College London, London, UK; 2 Mediteknia Skin and Hair Laboratory, Universidad Fernando Pessoa Canarias, Las Palmas de Gran Canaria, Spain

**Keywords:** Translational research, Regeneration

## Abstract

Despite the substantial impact of skin scarring on patients and the healthcare system, there is a lack of strategies to prevent scar formation, let alone methods to remodel mature scars. Here, we took a unique approach inspired by how healthy hairbearing skin undergoes physiological remodelling during the regular cycling of hair follicles. In this pilot clinical study, we tested if hair follicles transplanted into human scars can facilitate tissue regeneration and actively remodel fibrotic tissue, similar to how they remodel the healthy skin. We collected full-thickness skin biopsies and compared the morphology and transcriptional signature of fibrotic tissue before and after transplantation. We found that hair follicle tranplantation induced an increase in the epidermal thickness, interdigitation of the epidermal-dermal junction, dermal cell density, and blood vessel density. Remodelling of collagen type I fibres reduced the total collagen fraction, the proportion of thick fibres, and their alignment. Consistent with these morphological changes, we found a shift in the cytokine milieu of scars with a long-lasting inhibition of pro-fibrotic factors TGFβ1, IL13, and IL-6. Our results show that anagen hair follicles can attenuate the fibrotic phenotype, providing new insights for developing regenerative approaches to remodel mature scars.

## Introduction

Tissue remodelling is the reorganization of tissue architecture, which can be either physiological, responsible for directing the development and maintenance of tissues, or pathological, occurring after tissue injury^1^. During wound repair initiated by an injury to the skin, cells migrating into the wound bed deposit a mass of granulation tissue to re-establish the barrier integrity and prevent infection. The newly deposited tissue is then remodelled to restore the architecture and function of the skin. While this pathological remodelling can last for several months, and even years, the skin never truly regains the properties of the un-injured state^2^. Eventually, most cells within the fibrotic tissue undergo apoptosis or migrate away from the repair site, leaving a mass of incompletely remodelled tissue known as a scar^3^.

Scarring imposes an enormous burden on individuals and society, with an estimated 100 million people per year acquiring scars in high-income countries alone, primarily as a result of surgeries^3^. The global incidence of scars is much higher and includes extensive scarring formed after burn and traumatic injuries. Until now, all efforts to prevent scar formation or remodel fibrotic tissue yielded only suboptimal results. Traditional strategies to reduce scar formation include incisions along Langer’s lines, deep sutures to bring skin edges together, and dressings that offload tension from the wound^3^. More recently, various types of lasers, dermabrasion, and microneedling treatments have been assessed to improve scar appearance^3^. Despite their popularity, there have been no large-scale clinial studies to prove their effectiveness. Studies into the molecular mechanisms of fibrosis have yielded several novel targets that have been tested clinically, but the results have been largely disappointing^4^. These approaches include inhibiting cytokines and growth factors (PDGF, TGF-β1, CTGF)^5^, administration of TGF-β3, and modulating angiogenesis^6^. Following these studies, it has become clear that single-agent therapies based on secreted factors or their inhibitors are largely ineffective due to the complexity of the wound repair process and rapid protein degradation at the wound site^2^. To overcome this issue, effective anti-fibrotic therapy needs to be based on the long-term delivery of multiple factors to drive a sustained response that remodels the fibrotic tissue towards complete regeneration^7^.

In contrast to incompletely remodelled scar tissue, healthy skin undergoes constant physiological remodelling occurring during the growth stage of the hair follicle cycle^8^. During the cycle, hair follicles transition through growth (anagen), regression (catagen), and rest (telogen) stages. In mouse skin, follicles grow synchronously with one another, and as such whole areas of skin can contain hair follicles that are entirely in anagen, catagen or telogen at a time^9^. In mouse skin containing anagen hair follicles, the epidermis, dermis, and dermal white adipose tissue are between 1.6- and 2.0- fold thicker than the equivalent layers in skin containing telogen hair follicles^10^. Even though the dermis is thicker in skin with anagen hair follicles, the total number of cells in the dermis is the same as that observed in skin with telogen follicles^11,12^—this has led to the suggestion that extracellular matrix (ECM) redistribution facilitates changes to dermal thickness^13^. A similar parallel is observed with skin vasculature—angiogenesis and an extensive blood vessel network is observed around anagen follicles, yet this is diminished in catagen and telogen^14–17^. While hair follicle cycling and remodelling of interfollicular skin are clearly connected, the mechanism and extent by which hair follicles can regulate remodelling remains unclear^8^.

**Fig. 1:**
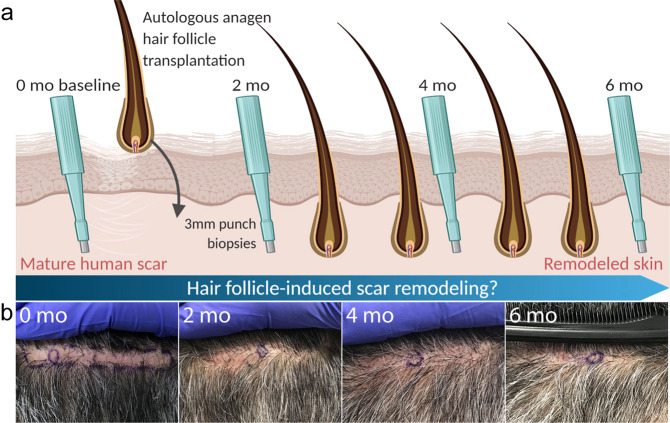
Schematic of the experimental outline and hypothesis. **a** We established a pilot clinical study whereby autologous anagen hair follicles were transplanted into mature scalp scars. We collected full-thickness skin biopsies of scars before (baseline at 0 months (mo)) and at 2, 4, and 6 months after hair follicle transplantation (2, 4, 6 mo). We compared the morphology and transcriptional signature of fibrotic tissue to test our hypothesis that anagen hair follicles can remodel mature scar tissue towards a healthy phenotype. Schematic created with BioRender.com. **b** We recruited three individuals with mature (at least 4 years old) normotrophic scars formed post-surgically on occipital scalps. Circles indicate areas where the full-thickness biopsies were taken.

To address the pressing need for an effective anti-fibrotic treatment, we took inspiration from observations of skin remodelling which occur during the growth stage of the hair follicle cycle in mice. We hypothesized that anagen hair follicles can remodel mature scars in human skin, similar to how they remodel healthy tissue in murine skin. We therefore took advantage of the routine procedure performed in hair transplantation clinics whereby anagen hair follicles are transplanted into scalp scars to camouflage the hairless fibrotic area formed as a result of previous hair transplantation surgeries, and designed a pilot clinical study to test this hypothesis in human skin (Fig. 1). To test if tissue remodelling is induced by anagen hair follicles transplanted into scars, we compared mature fibrotic tissue before (0 months) and after (2, 4, 6 months) hair follicle transplantation and found a shift towards the morphology and genetic profile of healthy skin. The results of this study lay the foundation for designing therapeutic strategies that dynamically remodel mature human scars and induce a long-lasting shift towards skin regeneration by mimicking the natural ability of hair follicles to remodel skin.

## Results

### Hair follicles transplanted into mature scars continue to grow

The stress of transplantation is known to induce hair follicles to enter catagen, followed by telogen, and new anagen^18^. Clinical and experimental evidence suggests that follicles re-enter anagen approximately 60 days post-transplantation and are in full anagen 90 days post-transplantation^19,20^. In our study, we took 3 mm full-thickness punch biopsies of scars before (timepoint 0 months as the baseline) and at 2, 4, and 6 months after hair follicle transplantation in 3 patients (Fig. 1a, Supplementary Fig. 1a, Supplementary Table 1). The clinical photographs at these timepoints show that transplanted hair follicles are producing hair fibres by the follow-up period (Fig. 1b, Supplementary Fig. 1a). We also stained 10 μm-thick sections of scar biopsies using haematoxylin and eosin (H&E) and found that all follicles were already in anagen by 2 months post-transplant (Supplementary Fig. 2), confirming previous observations^19,20^. As we hypothesized that anagen hair follicles would promote remodelling of fibrotic tissue, we next looked at interfollicular tissue in 2, 4 and 6 month biopsies and compared against the 0 month baseline.

### Anagen hair follicles remodel the epidermis of mature scars

Epidermal function is impaired in mature scars, which are prone to tears due to their thin epidermis and flat basement membrane with reduced collagen type IV (COLIV) expression^21,22^. We hypothesized that transplantation of anagen hair follicles into mature scars would induce an increase in the epidermal thickness and improve the interdigitation of the epidermal–dermal junction (EDJ).

**Fig. 2:**
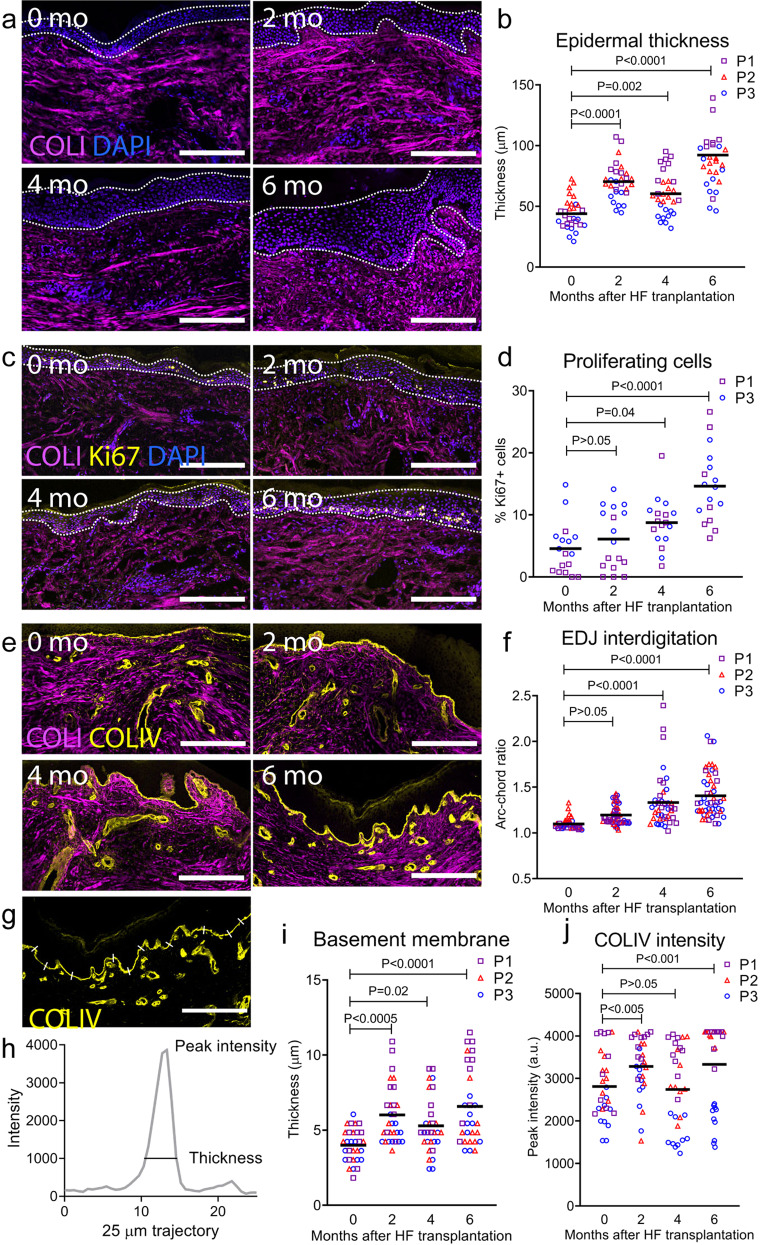
Anagen hair follicles remodel the epidermis of mature scars. **a** Representative immunofluorescence images of scars before (0 mo) and at 2, 4, and 6 months post-transplant of anagen hair follicles. We used DAPI to image cell nuclei and measure the thickness of the viable epidermis (dotted lines). **b** We observed an increase in the epidermal thickness after hair follicles (HF) were transplanted into mature scars (*n* = 20, *N* = 3). **c** Staining for Ki67 to quantify the percentage of proliferating cells in the viable epidermis (dotted lines) of mature scars before (0 mo) and after (2, 4, 6 mo) hair follicle transplantation. **d** The percentage of proliferating cells in the scar epidermis increased after hair follicle transplantation (*n* = 8, *N* = 2). **e** Epidermal–dermal junction (EDJ) stained for collagen type IV (COLIV) present in the basement membrane. **f** The arc-chord ratio revealed increased interdigitation after transplantation of hair follicles (*n* = 20, *N* = 3). **g** We measured the immunofluorescence signal of collagen type IV (COLIV) across the EDJ by probing the data along 25 µm trajectories traversing the EDJ. **h** The thickness of the basement membrane and abundancy of COLIV were measured as the width and peak of the COLIV intensity profiles. **i** The thickness of the basement membrane increased post-transplant as compared to mature scars (*n* = 20, *N* = 3). **j** The peak intensity increased at 2 and 6 months after transplantation (*n* = 20, *N* = 3). Reported *P* values are based on two‑way ANOVA tests, and the horizontal lines show grand means. Scale bars = 200 µm.

To test this, we imaged 10 μm-thick sections of scars before (0 months) and at 2, 4 and 6 months post-transplant at a minimum distance of 200 μm away from the transplanted hair follicles to ensure testing of the interfollicular scar tissue. We used 4′,6-diamidino-2-phenylindole (DAPI) nuclear counterstain to image cell nuclei and measure the thickness of the viable epidermis, which includes the basal, spinous and granular layers (Fig. 2a). Consistent with the increase in epidermal thickness observed in mouse skin during anagen^10^, we found that the scar epidermis was on average 1.6 times thicker just 2 months after hair follicle transplantation (*P* < 0.0001), 1.4 times thicker at 4 months (*P* = 0.002), and 2.0 times thicker at 6 months (*P* < 0.0001) post-transplant as compared to the mature scar before transplantation (0 months) (Fig. 2b). This increase brings the epidermal thickness to approximately 100 µm, which is like that observed in healthy occipital scalp skin (data not shown). To test if an increase in epidermal thickness was accompanied by an increase in cell proliferation, we stained scar sections to detect the expression of Ki67, a marker of proliferating cells (Fig. 2c). We decided to exclude samples from patient 2 (P2) in this analysis due to technical challenges of identifying Ki67+ proliferating cells across all samples after an equipment malfunction, to avoid false negative data points. Based on data from P1 and P2, we found an average 4% of proliferating cells in the baseline scar epidermis, which increased after hair follicle transplantation to 9% at 4 months (*P* = 0.04) and 15% at 6 months (*P* < 0.0001)(Fig. 2d). Although we detected no significant difference between the percentage of proliferating cells in the epidermis before transplantation and at 2 months post-transplantation (*P* > 0.05), we need to consider that we quantified the proliferation of cells at a snapshot in time (2 months) while the observed increase in the epidermal thickness is likely a result of 2 months of accumulated increased proliferation. Compared to previous reports on normotrophic scars^23^, scalp scars in our study presented a lower percentage of Ki67-positive proliferating cells in the epidermis (4%), which increased to the levels of other normotrophic scars (17%) and normal skin (16%) at 6 months post-transplant (15%). This large difference in the baseline level of proliferating cells (17% vs 4%) between normotrophic scars and those in our study can be explained by the maturity (1 year vs 4 years old) and location (abdomen, neck, back versus scalp) of studied scars^23^.

To evaluate the effect of anagen follicles transplanted into mature scars on the EDJ, we used staining for COLIV present in the basement membrane (Fig. 2e). We quantified the amount of interdigitation by measuring the arc-chord ratio of the curve defining the EDJ (Fig. 2f). Although we observed no change in EDJ interdigitation at 2 months post-transplant (*P* > 0.05), we found that EDJ interdigitation increased at 4 (1.2-fold, *P* < 0.0001) and 6 (1.3-fold, *P* < 0.0001) months compared to the 0 month baseline (Fig. 2f). The thickness of the basement membrane and abundancy of COLIV were quantified based on the COLIV fluorescence intensity profile across the EDJ (Fig. 2f). We observed an average 1.5-fold increase (*P* < 0.0005) in basement membrane thickness at 2 months, followed by 1.3-fold (*P* = 0.02) and 1.6-fold (*P* < 0.0001) increases at 4 and 6 months post-transplant (Fig. 2i). The peak intensity of COLIV expression was higher at 2 (1.2-fold, *P* < 0.005) and 6 months (1.2-fold, *P* < 0.001) post-transplant compared to the 0 month baseline (Fig. 2j).

Collectively, these results lend support to our hypothesis that the initially thin epidermis and flat basement membrane of mature scars undergo remodelling upon transplantation of anagen hair follicles.

### Anagen hair follicles remodel the dermis of mature scars

**Fig. 3:**
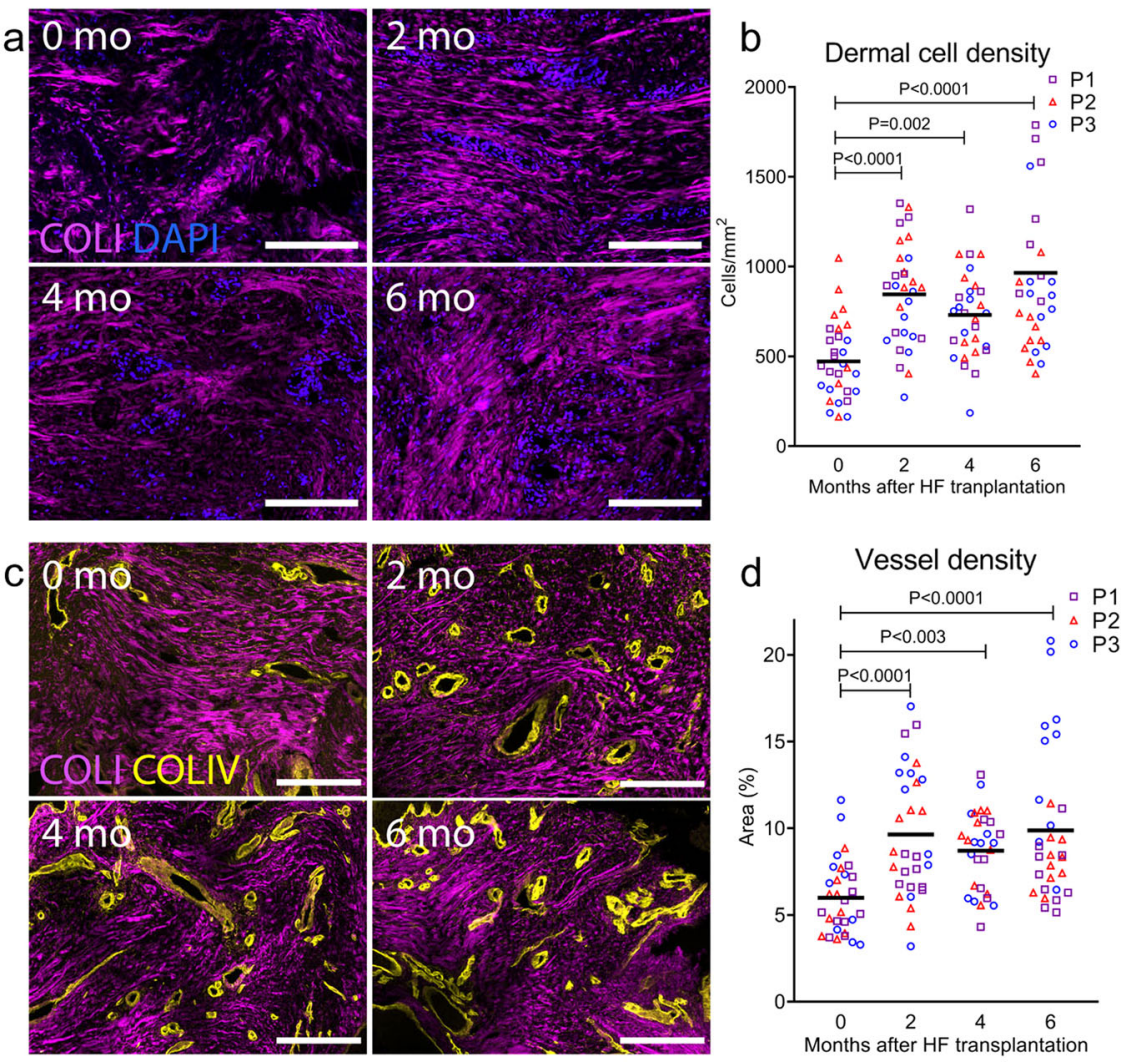
Anagen hair follicles remodel the dermis of mature scars. **a** Representative immunofluorescence images of 10 μm-thick sections of mature scars before (0 mo) and at 2, 4, and 6 months post-transplant of anagen hair follicles. We used DAPI nuclear counterstain to quantify cell nuclei within the dermis and second harmonic generation (SHG) imaging for collagen type I (COLI) fibres. **b** Quantification of DAPI-stained nuclei revealed an increase in the dermal cell density at 2, 4, and 6 months after hair follicle (HF) transplantation (*n* = 20, *N* = 3). **c** Immunofluorescence imaging of scar sections for collagen type IV (COLIV) present in the basement membrane of blood vessels combined with SHG imaging. **d** We quantified the area (%) covered by COLIV staining and found that the dermis of scars contains higher vessel density after hair follicle transplantation (*n* = 20, *N* = 3). Reported *P* values are based on two‑way ANOVA tests, and the horizontal lines show grand means. Representative images from one patient, scale bars = 200 µm.

The skin dermis contains a network of dense ECM populated with cells^22^. After skin injuries that are sufficiently deep to destroy the root of the hair follicle, there is an accumulation of incompletely remodelled ECM lacking hair follicles (a scar)^24^. During scar maturation, fibroblasts decrease in number resulting in reduced dermal cell density. Scar tissue is also characterized by a lower amount of cutaneous fat, possibly caused by the lack of hair follicles that would normally stimulate transdifferentiation of myofibroblasts into adipocytes^25^. To test if transplantation of anagen hair follicles into mature scars can reverse this process, we used DAPI nuclear counterstain to image cell nuclei within the dermis, combined with second harmonic generation (SHG) imaging to visualize collagen type I (COLI) fibres (Fig. 3a). Dermal cell density in mature scars after follicle transplantation increased on average 1.8-fold at 2 months (*P* < 0.0001), 1.5-fold at 4 months (*P* = 0.002), and 2.0-fold after 6 months (*P* < 0.0001), compared to the 0 month baseline (Fig. 3b). This increase brings the dermal cell density to approximately 1000 cells/mm^2^, which is like that of healthy occipital scalp skin (data not shown). We were unable to perform a comparative analysis and make conclusions about the adipose tissue due to the variations in the depth at which biopsies of the interfollicular skin were taken.

Morphological changes to the dermis after skin injuries are also reflected in the extent of vascularization. Capillaries, which are initially formed in the proliferative stage of the wound healing process, degenerate during remodelling leaving mature scars with low vascularization^24^. To test if anagen hair follicles transplanted into mature scars can stimulate angiogenesis, we compared vessel density before and after hair follicle transplantation using staining for collagen type IV (COLIV) present in the basement membrane of blood vessels (Fig. 3c). The greatest average vessel density of 10% was observed at 2 (*P* < 0.0001) and 6 months (*P* < 0.0001) after hair follicle transplantation as compared to the mature scar before transplantation (6%), with a vessel density of 9% at 4 months (*P* = 0.003) (Fig. 3d). Average vessel density in healthy occipital scalp skin is approximately 10% (data not shown), so this observed increase makes the vessel density similar to that found in healthy scalp dermis.

Our findings so far demonstrate that transplantation of anagen hair follicles into mature human scars can reverse the decrease of dermal cell density and vascularization that occur during scar maturation. Consistent with the epidermal remodelling observed after hair follicle transplantation, these results support the hypothesis that anagen hair follicles can induce long-lasting remodelling changes to the fibrotic tissue.

### Anagen hair follicles remodel the architecture of COLI in mature scars

Scar tissue is composed of incompletely remodelled ECM, predominantly composed of COL1 fibres. To see if anagen hair follicles can induce remodelling of the COLI fibres, we used SHG microscopy and a method for collagen analysis based on an interactive learning and segmentation software ilastik (see Methods and Supplementary Fig. 3a).

**Fig. 4:**
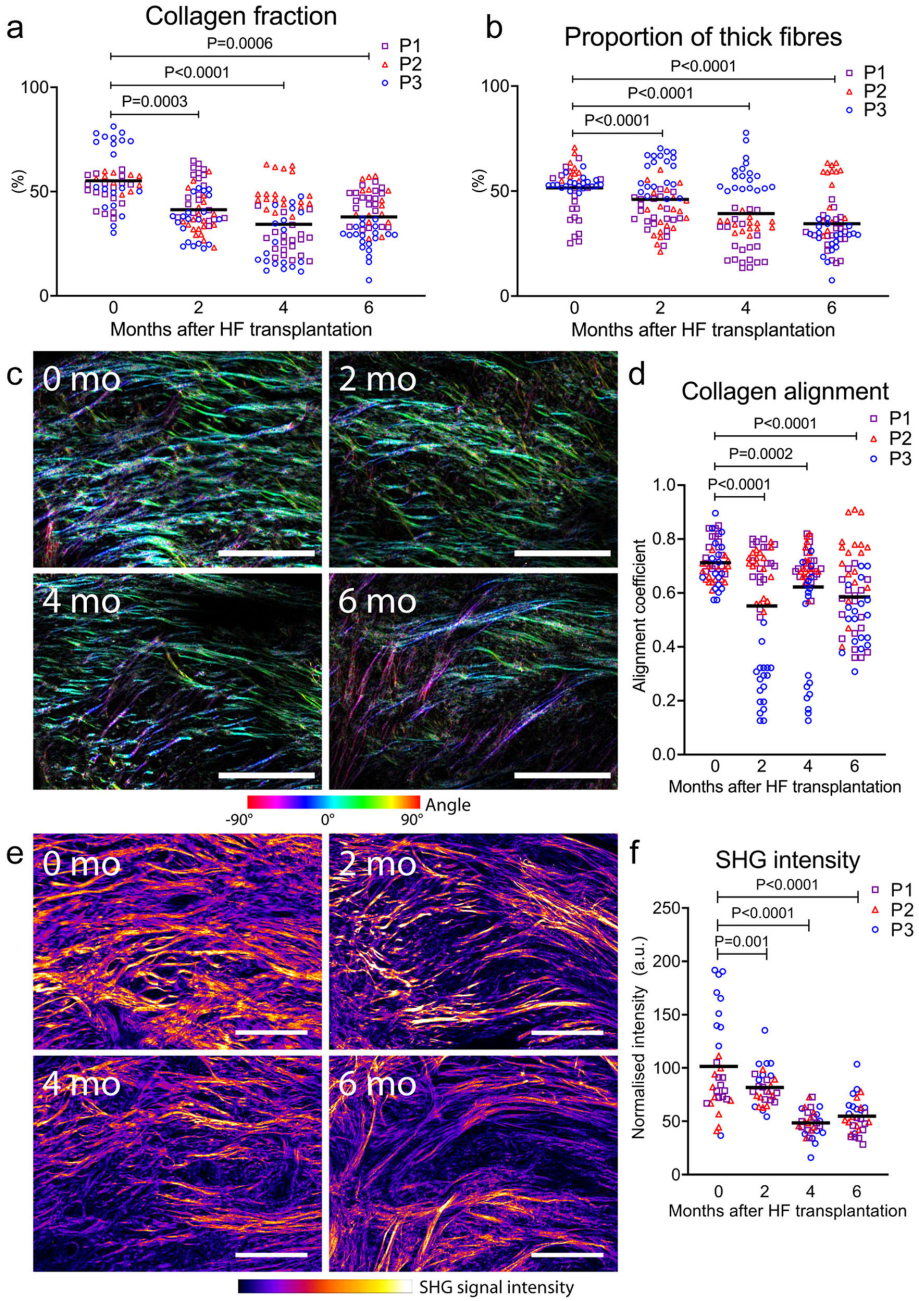
Anagen hair follicles remodel the architecture of COLI in mature scars. **a** Analysis of second harmonic generation (SHG) images reveals a decrease in the total fraction of collagen type I (COLI) fibres and (**b**) proportion of thick fibres at 2, 4, and 6 months after hair follicle (HF) transplantation into mature scars (*n* = 36, *N* = 3). (**c**) Colour-coded maps of fibre orientation show preferential alignment of fibres along the epidermis (0° angle) before hair transplantation. (**d**) At 2 months post-transplant, collagen fibres become more disorganized as the alignment coefficient decreases (*n* = 18, *N* = 3). **e** The SHG signal intensity can be used as a proxy measurement for tension in collagen fibres. **f** We found that the SHG signal intensity decreases at 2 months after hair follicle transplantation and remains low throughout the follow-up period (*n* = 20, *N* = 3). Reported *P* values are based on two‑way ANOVA tests, and the horizontal lines show grand means. Representative images from one patient, scale bars = 200 µm.

Since mature scar ECM consists of a greater density of thicker COL1 fibres compared to healthy skin^26^, our analysis focused on the total collagen fraction and the proportion of thick fibres. We found an average 1.3-fold decrease in the total collagen fraction after 2 months (*P* = 0.0003), which was persistent at 4 months (1.6-fold, *P* < 0.0001), and 6 months (1.5-fold, *P* = 0.0006) after hair follicle transplantation (Fig. 4a). Similarly, the proportion of thick collagen fibres was initially reduced from an average of 52% in mature scars, to 46% at 2 months (*P* < 0.0001), 39% at 4 months (*P* < 0.0001), and 35% at 6 months post-transplant (*P* < 0.0001) (Fig. 4b).

In mature scars, instead of a normal basket-weave pattern of collagen fibres found in healthy skin, collagen forms cross-links that induce alignment in a single direction parallel to the EDJ^27^. As a result of increased fibre orientation and density, scar dermis increases in stiffness from 0.75 MPa of un-injured skin to 3 MPa during scarring^28,29^. To assess fibre orientation in scars after hair follicle transplantation, we created colour-coded maps based on SHG images and plotted graphs of fibre orientation (Fig. 4c and Supplementary Fig. 3b). The preferential orientation of fibres in mature scars parallel to the epidermis (at 0°) changes already at 2 months post-transplant, when collagen fibres become more disorganized as indicated by a decreased collagen alignment coefficient (*P* < 0.0001) (Fig. 4c) and a higher spread of orientation angles (Supplementary Fig. 3b). Since tissue with highly aligned collagen fibres is stiffer when loaded parallel to the fibre direction, the reduced fibre organization in mature scars post-transplant suggests decreased tissue stiffness^30^.

The level of pretension on COLI fibres can also determine the mechanical properties of the skin by affecting its ability to resist deformation^31^. We therefore used the SHG signal intensity as a proxy measure of tension on collagen fibres (Fig. 4e)^32^ finding it decreased on average by 1.2-fold (2 months), 2.1-fold (4 months), and 1.9-fold post-transplant (*P* < 0.001) (Fig. 4f). These results suggest that reduced tension on dermal collagen fibres after hair transplantation would lead to fibrotic tissue that can tolerate higher stresses.

This data reports changes to the morphology of COLI fibres, including reduced collagen fraction, the proportion of thick fibres, and lower fibre alignment after hair follicle transplantation, suggesting a regenerative shift towards the phenotype of un-injured skin.

### Anagen hair follicles remodel a split-thickness skin graft scar

The above analyses demonstrate remodelling of the epidermis and dermis of mature scalp scars after transplantation of anagen hair follicles. However, the remodelling effect from the transplanted hair follicles could be influenced by the presence of a high density of anagen hair follicles surrounding scalp scars. To overcome this bias, we sought to test the effect of anagen hair follicles transplanted into a mature scar formed on a body site with a lower density of surrounding hair follicles in anagen (Supplementary Fig. 1b, Supplementary Fig. 2b and Supplementary Table 1). For this we used a split-thickness skin graft (STSG) scar, and found significant increases in epidermal thickness, EDJ interdigitation, dermal density, vascularization, and collagen remodelling by the 2 month timepoint (Supplementary Figs. 4–7) as compared to the 0 month baseline. The changes to collagen architecture were not maintained at 6 months post-transplantation indicating a more transient regeneration induced by hair follicles in this type of scar (Supplementary Figs. 4–7).

### Anagen hair follicles change the transcriptional signature of mature scars

**Fig. 5:**
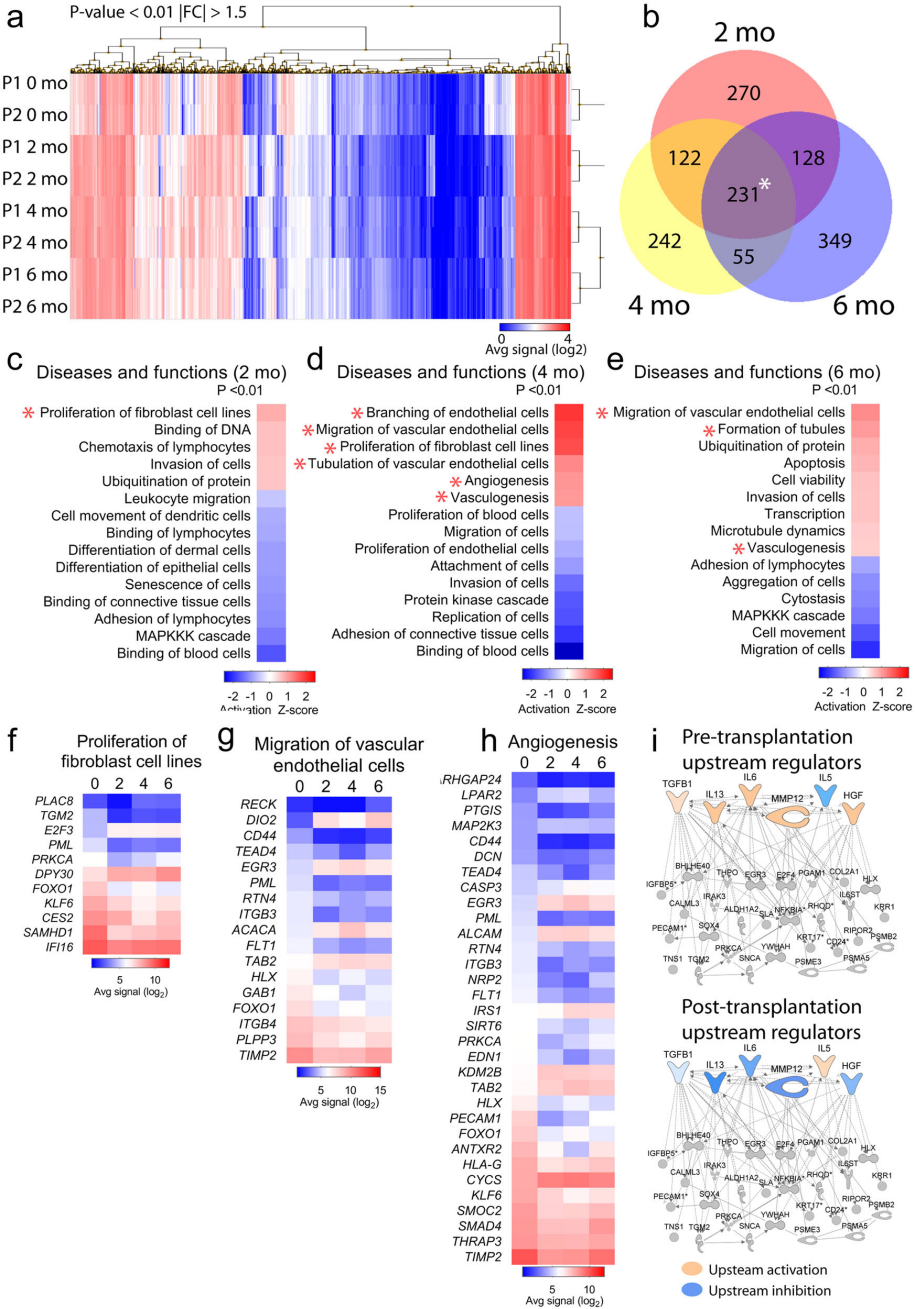
Anagen hair follicles change the transcriptional signature of mature scars. **a** We used Transcriptomic Analysis Console to perform hierarchical clustering analysis on 719 genes that were differentially (|FC| > 1.5) and significantly (*P* < 0.01) regulated in the scar dermis before (0 mo) and after (2, 4, 6 mo) hair follicle transplantation. |FC| = absolute fold change (**b**) Venn diagram showing the transient transcriptomic changes to the scar dermis occurring post-transplant as compared to the baseline (0 mo). Asterix marks the core transcriptional signature which consists of 231 transcripts that were differentially regulated already at 2 months post-transplant and remained so throughout the follow-up period. **c**–**e** Ingenuity Pathway Analysis (IPA) analysis highlighting function terms in differentially regulated genes in the scar dermis after hair follicle transplantation. We selected functions terms that were relevant to the skin and had an |activation z-score| > 0.5. **f**–**h** Average signal of genes involved in functions terms relevant for the study, detected in the scar dermis before (0 mo) and after hair follicle transplantation (2, 4, 6 mo). **i** Downstream transcriptional network of secreted upstream regulators (*P* < 0.05) of the dataset of 231 core signature genes.

Our histological analyses showed that anagen hair follicles transplanted into mature scalp scars induce morphological changes to the fibrotic tissue towards the healthy skin phenotype. To identify gene expression patterns underlying this regenerative signature we performed an unbiased transcriptomic analysis using Affymetrix microarrays on the skin dermis isolated from the 0, 2, 4 and 6 month samples. Using Transcriptomic Analysis Console, we performed a one-way ANOVA analysis on the dataset and identified 719 genes that were differentially (|fold change| > 1.5) and significantly (*P* < 0.01, FDR < 0.01) regulated after hair follicle transplantation into mature scars (Fig. 5a). Hierarchical clustering analysis of the differentially expressed genes demonstrated that the transcriptional signature of later-stage remodelling (4 and 6 mo post-transplant) are most similar to one another (Fig. 5a). We used total RNA isolated from the scar dermis of the third patient involved in the study but not included in the microarray analysis to validate the expression changes of selected transcripts (Supplementary Fig. 8). Despite slight variations in the gene expression patterns between the PCR and microarray results, which need to be considered in the context of high heterogeneity of human samples, our data provide a strong validation for the microarray results. To identify transient transcriptomic changes occurring in the scar dermis, we compared the scar transcriptome at each timepoint after hair transplantation against the baseline before transplantation [0 vs 2, 0 vs 4, 0 vs 6 months)], identifying 270 genes that were expressed uniquely at 2 months, 242 genes at 4 months, and 349 genes at 6 months post-transplant (Fig. 5b). Using these gene lists, we performed a core analysis based on the fold change expression using the Ingenuity Pathway Analysis software (IPA, Qiagen) and identified diseases and functions terms that are relevant to the skin and have an |activation z-score| > 0.5 (Fig. 5c–e). Among the terms detected in the scar dermis after transplantation, we found transcripts involved in fibroblast proliferation (Fig. 5c, f), explaining the histological observation of increased dermal cell density (Fig. 3b). An upregulation of terms and transcripts involved in the migration of vascular endothelial cells (Fig. 5d, g) and angiogenesis (Fig. 5d, h) supports the finding of increased vascularization in the scar dermis after hair transplantation (Fig. 3d).

Besides the transient transcriptomic changes to the scar dermis occurring at different timepoints post-transplant, there was also a core transcriptional signature consisting of 231 transcripts that were differentially regulated already at 2 months post-transplant and remained so throughout the follow-up period (Fig. 5b). Due to previous reports on the role of hair follicle secretome in both physiological and pathological skin remodelling^33,34^, we hypothesized that hair follicles might contribute to scar remodelling via paracrine signaling. To identify changes to the cytokine milieu of mature scars after hair follicle transplantation, we performed another analysis of the core transcriptional signature (231 transcripts) using the IPA software and selected all secreted upstream regulators (*P* < 0.05) (Supplementary Table 2). We postulate that paracrine factors from the hair follicle leads to activation or inhibition of these regulatory factors within the scar dermis. To predict their regulatory direction, we used MAP (Molecule Activity Predictor) by overlying measurement values from a dataset of differentially expressed genes at 2 months post-transplant compared to the 0 month baseline. Using this approach, we predicted a long-lasting inhibition of transforming growth factor beta 1 (TGF-β1), hepatocyte growth factor (HGF), interleukins (IL-13, IL-6), and matrix metalloproteinase 12 (MMP-12), and activation of IL-5, in the scar dermis after hair follicle transplantation (Fig. 5i). Among these regulators, TGF-β1, IL-13 and IL-6 have been previously identified as pro-fibrotic factors^7^, while the role of MMP-12 and HGF in fibrosis remains context-dependent and is not well described in the skin^35,36^. Of the known pro-fibrotic regulators identified, both TGF-β1 and IL-6 decrease in expression in the scar dermis after transplantation of hair follicles (Supplementary Fig. 8). They are both also expressed by dermal fibroblasts (Supplementary Fig. 9a, Supplementary References). To evaluate the short-term effect of hair follicles on TGF-β1 and IL-6 expression, we co-cultured fibroblasts with hair follicles for 7 days, then evaluated expression of TGF-β1 and IL-6. There was a significant decrease in expression of TGF-β1 in fibroblasts co-cultured with hair follicles, and a significant increase in the expression of IL-6 (Supplementary Fig. 9b). While the observed decrease of TGF-β1 is supportive of the predicted inhibition after the addition of hair follicles, the increase in expression of IL-6 counters this view. This discrepancy can be explained by the differences between a short-term (in vitro) assay and the longer term *(*in vivo*)* assay on which the MAP analysis was performed.

The results so far suggest that transplantation of anagen hair follicles into scar tissue can lead to tissue remodelling towards a healthy phenotype. However, the effect of transplantation in terms of the physical protrusion created by the introduction of a follicle and the subsequent wound response cannot be negated. To evaluate our hypothesis that hair follicles induce remodelling as appose to the response elicited by their transplantation, we needed to move to a ‘clean’ wound free system. As TGF-β1 expression was decreased in fibroblasts after the addition of hair follicles, and decreased in scar dermis after hair follicle transplantation, we decided to assess the effect of hair follicles on scar formation in an in vitro scar model induced by culturing skin fibroblasts in the presence of TGF-β1^37^. When hair follicles were introduced to this model but separated from the developing in vitro scar by a transwell membrane, we found significantly reduced expression of ECM molecules, including collagen type I (COLI), collagen type III (COLIII), ɑ-SMA, and fibronectin (Supplementary Fig. 10). This data supports our hypothesis that hair follicles influence ECM deposition via paracrine signalling mechanisms, and that the observed scar remodelling after hair follicle transplantation is not a secondary effect of wound healing.

Collectively, our results show that anagen hair follicles transplanted into mature scars can alter the fibrotic transcriptional signature. The predicted changes to the cytokine milieu of fibrotic tissue demonstrate the complexity of the long-term transcriptional response within human scars after transplantation of anagen hair follicles. Unlike single-agent anti-fibrotic therapies causing only transient changes to selected molecular pathways, hair follicles induce a long-term global change to the scar transcriptome, affecting multiple cell types and cellular functions.

## Discussion

**Fig. 6:**
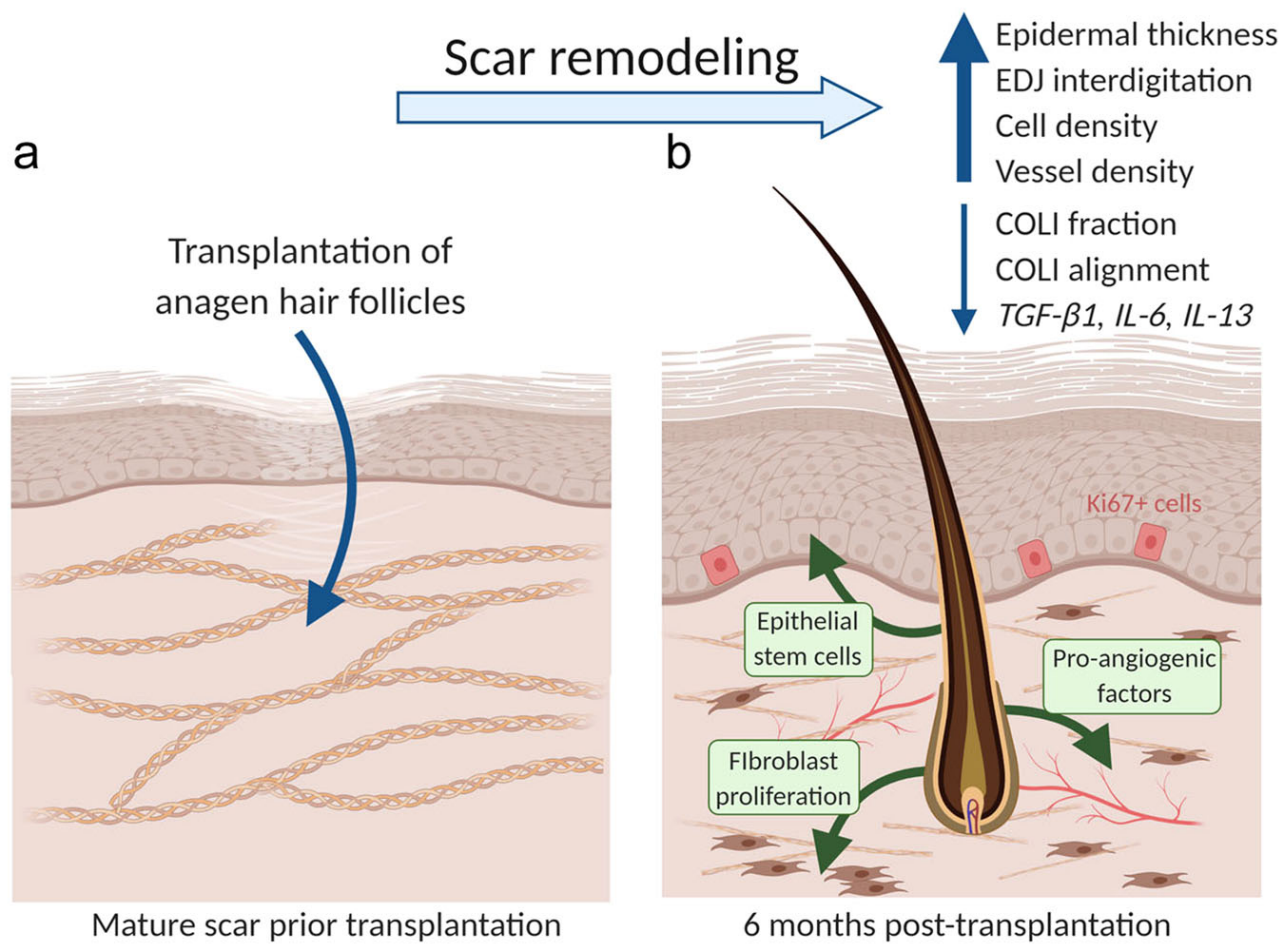
Summary of results and the proposed mechanisms. **a** Mature human scars are characterized by thin epidermis, flat basement membrane, hypocellular environment with low vessel density and dense, thick, and highly aligned collagen type I fibres. In our study, we tested if anagen hair follicles transplanted into mature scars can remodel fibrotic tissue, the same way they remodel the healthy skin during the natural hair follicle cycle. **b** After autologous anagen hair transplantation into mature scars, we found an increase in the epidermal thickness and interdigitation of the epidermal-dermal junction, which we propose to be caused by migration of epithelial stem cells from the hair follicle. The increase in dermal cell and vessel densities could be caused by secretion of remodelling growth factors from the transplanted hair follicles. Remodelling of COLI fibres post-transplant reduced their total fraction, thickness, and alignment, and could be caused by a yet unknown direct effect from the hair follicle. These morphological changes towards the healthy skin phenotype were consistent with changes to the transcriptional signature showing a long-lasting inhibition of pro-fibrotic cytokines TGF-β1, IL-13, and IL-6. Schematic created with BioRender.com.

The physiological endpoint of wound repair in humans leads to an accumulation of incompletely remodelled fibrotic tissue known as a scar. Multiple strategies have been tested to minimize scarring, but despite years of efforts, there is no effective method to remodel mature scars^3^. In this body of work, we sought to identify new potential avenues for developing active therapies by taking inspiration from healthy hairbearing skin that undergoes remodelling during the growth stage of the hair follicle cycle (anagen). We hypothesized that anagen follicles could promote scar remodelling and tested this in a pilot clinical study on mature human scars. Using skin scars as an accessible model for fibrosis, we found that transplantation of anagen hair follicles leads to active remodelling of fibrotic tissue towards a healthy skin phenotype, inducing changes to the morphology and transcriptional signature of mature scars (Fig. 6a, b). We discuss both the results and limitations of our study here.

While previous work explored the role of anagen hair follicles in wound closure^38,39^, to our knowledge, this current study is the first to test the ability of hair follicles to alter the morphology and transcriptional signature of scar tissue created after the wound has closed. A classic study from 1945 demonstrated that in shallow cutaneous wounds in humans, re-epithelialization starts around the remaining hair follicles^40^ while lineage tracing in mice has now shown this to be through migration of epithelial stem cells out of the hair follicle^41^. Here, we found that hair follicles transplanted into scars induced an increase in the epidermal thickness and interdigitation of the EDJ, and hypothesize that this occurs through the same migratory mechanism that facilitates wound healing (Fig. 6b). Furthermore, anagen hair follicles grafted into chronic wounds can accelerate wound closure by enhancing re-epithelialisation^42^, and increasing the number of blood vessels and lymph vasculature in the skin dermis^43^. In our study looking at scar remodelling, we found increased cell density and vascularization post-transplant, and a reduction in the total fraction of COLI fibres and the proportion of thick fibres (Fig. 6). This can be explained by either migration of fibroblasts out of hair follicles to support dermal remodelling, or the release of remodelling factors directly from the follicle (Fig. 6b). Since no increase in the dermal cell density has been previously reported as part of the physiological remodelling during anagen^44^, we propose that it is paracrine signalling from hair follicles that leads to remodelling of the fibrotic dermis. The mechanism could include release of pro-angiogenic VEGF from the hair follicle components^16,45,46^, or EGF that is known to be involved in both the hair follicle cycle and fibroblast proliferation^47,48^.

A phenomenon of wound-induced hair neogenesis (WIHN) provides further evidence for the link between hair follicle growth and skin regeneration. In the mouse model of WIHN, large-size excisional skin wounds can heal by activating spontaneous regeneration of new hair follicles in the wound center^49^. Thus, WIHN provides a naturally occurring mouse model that is analogous to hair-grafted scars. We are not aware of any studies to date that have compared the morphology and transcriptomic profile of scar tissue with and without WIHN, although it would be intriguing to see if results from these studies would corroberate our observations in human skin.

Our analysis of scar remodelling was limited by clinical constraints that restricted the size, number, and timing of skin biopsies. Our study focused on long-term scar remodelling with a follow-up period of 6 months post-transplant and the number of timepoints was determined by taking into consideration the comfort of the subjects. The morphological changes that occurred in scars after hair follicle transplantation, including increased epidermal thickness and vessel density, resemble the wound healing process^2^. Performing a sham transplantation with non-hairy skin would eliminate the bias of inducing wound healing. However, such intervention was not possible due to ethical limitations given that patients involved in the study were undergoing aesthetical hair transplantation surgeries. In such study, the analysis of scar tissue 200 µm away from the transplanted hair follicle would also not be possible, which was a distinctive aspect of our study design. To circumvent this limitation, we instead aimed to assess if the underlying transcriptomic changes involved in scar remodelling that we observed after hair follicle grafting had any similarities to skin response to injury and searched the Gene Expression Omnibus (GEO) database for a control dataset. However, we could not identify any other study on scar remodelling that provided transcriptomic data from an in vivo evaluation of human skin. The lack of such findings stresses the need for studies like ours, providing novel transcriptomic data and insights to the research community. In addition, we know of no study that suggests that creating a small number of sparse wounds in the dermis would elicit scar remodelling to the extent observed in our work. Despite a limited number of subjects, our study had sufficient power to detect significant morphological and transcriptional differences in fibrotic tissue before and after hair transplantation.

It has been controversial whether advanced fibrosis can be reversed to the extent that the architecture and function of un-injured skin are fully restored^7^. Attempts to use single-agent therapies to improve scar morphology have been disappointing, likely due to the limited impact a single agent can have on a complex, long-term biological process such as scarring. In our study, the morphological shift of the scar dermis towards the healthy phenotype was consistent with the long-lasting changes to the cytokine milieu of fibrotic tissue showing an inhibition of pro-fibrotic cytokines TGF-β1, IL-13, and IL-6 in the skin dermis. As a result, the scar remodelling achieved through our strategy provides compelling support for the hypothesis that the delivery of multiple growth factors, cytokines, or their inhibitors, can have a long-lasting impact on the morphology and gene expression profile of mature scars. These findings provide exciting insights that can potentially seed a bioinspired anti-fibrotic strategy aiming to recapitulate the important features of the hair follicle that drive the remodelling of the fibrotic tissue.

We also conducted a study in one patient where scalp hair follicle grafts were transplanted into a STSG, and in this case only observed a transient remodelling effect of transplantation (see Supplementary Results). From our (FJ) experience, hair follicles transplanted into STSG scars have a survival rate of 70–90%, which is similar to that of secondary scarring alopecia^50–52^. This is also comparable to the survival rate (80–90%) of follicle grafts transplanted into strip scars^53^. This suggests that the duration of remodelling does not relate to the survival of hair follicles transplanted into the scars. A key difference between strip scars and STSG is the density and size of hair follicles in the surrounding skin. We believe a quorum sensing response in the surrounding skin may facilitate continued remodelling in the strip scar, but not the STSG where there are no surrounding hair follicles.

In our study involving scars formed on occipital scalps, the presence of hair after the transplantation was desirable. However, before the results of this study can be applied to achieve scar remodelling on non-hairy sites, further work is needed to understand the complex interactions occurring in the fibrotic tissue after hair follicle transplantation. A potential approach involves deciphering the type of hair follicle cells that contribute to scar remodelling and injecting them directly into scar tissue on non-hairy areas of the body. Alternatively, another approach would involve identifying the upstream paracrine factors produced by cells within the follicle that lead to the long-term inhibition TGF-β1, IL-13, and IL-6 in skin dermis. Such biological insights and clinical protocols will allow us to achieve skin regeneration without requiring transplantation of hair follicles into scars.

In closing, in this body of work we have presented morphological and transcriptional evidence that the transplantation of anagen hair follicles serves as a promising approach to remodel mature human scars, a long-standing clinical challenge. The ability to drive long-lasting changes to established fibrotic tissue through paracrine signalling opens up intriguing possibilities for new regenerative approaches. Ultimately, we hope this work will inspire therapeutic strategies designed to capture the combinatorial signaling that likely underpins the ability of hair follicles to actively remodel fibrotic tissue, in turn bringing us one step closer to impactful therapies for fibrotic tissue.

## Methods

### Study design

The goal of this study was to test the hypothesis that transplantation of anagen hair follicles promote remodelling of mature human scars. We focused on the most commonly occurring normotrophic scars and excluded keloid and hypertrophic scars of more complex pathology^54^. According to clinical evidence, repaired skin continues remodelling up to 2 years after injury. We therefore established a pilot clinical study involving 3 male patients with mature (at least 4 years old) and wide (at least 5 mm) scars formed post-surgically on occipital scalps after a Follicular Unit Transplantation (FUT) surgery (Fig. 1a, Supplementary Fig. 1a, Supplementary Table 1). After this surgery, most patients heal with narrow scars while a small number of patients (14.7%) heal with post-operating scar widening^55^, which we used in our study. An additional patient with a split-thickness skin graft (STSG) scar on the thigh was recruited to validate our findings (Supplementary Fig. 1b). An equal proportion of follicular units with 1, 2, and 3 hair fibres were first harvested from the donor site (occipital scalp) with 1 mm punch biopsies by means of the Follicular Unit Extraction (FUE) technique and cleaned from the attached adipose tissue. Follicular units with all hairs in anagen were selected and transplanted into scars at a density of approximately 15–20 follicular units per cm^2^. To quantify tissue remodelling induced by hair follicles, we collected 3 mm full-thickness punch biopsies of scars from a different location each time at 2, 4, and 6 months after hair follicle transplantation (Fig. 1b, Supplementary Fig. 1). Biopsies of scars collected before transplantation (0 months) served as a baseline control. Excised biopsies were embedded in OCT (optimal cutting temperature) medium and stored at −80 °C until needed. In addition to biopsy samples, clinical photographs of the skin were taken at baseline timepoint 0, then again at 2, 4 and 6 months.

### Study approval

Written informed consent was obtained from all patients after the nature and possible consequences of the study have been explained. All relevant ethical regulations for work with human participants were complied with. The clinical study was approved by the Research Ethics Committee at the University of Las Palmas de Gran Canaria (approval number CEIH-2017-17). The collection of human hair follicles and reticular fibroblasts used in the in vitro scar model was approved by the Joint Research Compliance Office at Imperial College London (ICREC reference: 17IC3726).

### Second harmonic generation (SHG) imaging and image analysis

Biopsies taken at four timepoints (0, 2, 4, and 6 months) were sectioned (100 µm sections), for second harmonic generation (SHG) imaging. The advantage of SHG over conventional immunochemistry for studying collagen type I (COL1) fibres include no need for labelling, high signal specificity, and high signal-to-noise ratio^56,57^. The imaging was performed on an upright confocal microscope (Leica SP5) coupled to a Ti:Sapphire laser (Mai Tai, Newport Spectra Physics), a water-immersion 25X objective (IRAPO, NA 0.95, Leica). A hybrid detector (HyD) detector was used to obtain a stronger SHG signal. The microscope parameters, including gain, accumulation, and averaging, were kept constant between imaging different samples.

To analyze the architecture COL1 fibres in the dermis, regions of interest (ROIs) of 600 μm × 450 μm were selected at a minimum distance of at least 200 μm away from the transplanted hair follicles to ensure testing of the interfollicular scar tissue from tile scans of approximately 3000 μm × 2000 μm. We used a pixel classification software ilastik that has previously been used for collagen analysis^58^. We trained the ilastik software on a set of six images to define pixels as belonging to one of the three features: thin fibres, thick fibres, and background (Supplementary Fig. 3). A probability mask for the segmentation of collagen fibres was subsequently applied to 20 images (single *Z* -stack steps) in an automatized batch processing mode. We used the Fiji Macro Recorder to automate the separation of segmented channels, quantification of the pixel area covered by each of the three features (thin fibres, thick fibres, and background), and measurement of the average fibre thickness with BoneJ plugin for ImageJ^59^. The fibres were clearly separated into thin fibres with an average width along its length ≤10 μm and thick >10 μm fibres. Based on the percentage pixel area covered by each of the three features, the total collagen fraction, and the proportion of thick collagen fibres were calculated.

The orientation of collagen fibres was assessed using an open source MATLAB software CurveAlign^60^. We used the same ROIs of 600 μm × 450 μm selected from tile scans as used for the assessment of proportion and thickness of collagen fibres. The primary settings with fraction of coefficient to keep = 0.06 and distance from boundary to evaluate = 150 pixels were used. The alignment of fibres with respect to each other was quantified as an alignment coefficient that ranges from 0 to 1, with 1 representing perfectly aligned fibres, and smaller values indicating fibres distributed more randomly. To measure the SHG intensity, the average image intensity was calculated using only pixels where fibres were present to ensure that the density of fibres did not bias the intensity evaluation. Normalized intensity was measured by dividing average intensity of ROIs by the average intensity of the background signal.

### Immunofluorescence and image analysis

OCT-embedded scar samples were cryosectioned at 10 µm, fixed with 4% paraformaldehyde (AGR1026, Agar Scientific) in phosphate-buffered saline (PBS) for 10 min at room temperature (RT). Tissue was permeabilized with 0.3% Triton X-100 (Sigma-Aldrich) at RT for 30 min, blocked with 5% goat serum and 5% donkey serum (Vector Laboratories) at RT for 30 min, then incubated with primary antibodies diluted in PBS (Supplementary Table 3) overnight at 4 °C. The sections were next washed three times in PBS and incubated in secondary antibodies (Supplementary Table 4) diluted in PBS for 1 h at RT in the dark. After three washes in PBS, sections were mounted using Vectashield mounting media and a cover slide. The excitation was accomplished at wavelength 430–480 nm for multiphoton signal from the DAPI nuclear counterstain. Through sequential scanning, separated excitation of 594 nm and emission 500–580 nm were used for fluorescence imaging of collagen type IV (COLIV). High resolution Z-stack images (1024 × 1024 pixels) with 12-bit pixel depth were obtained. To analyze images, ROIs were selected at a minimum distance of 200 μm away from transplanted hair follicles to ensure testing of testing of the interfollicular scar tissue. To analyze the morphology of scar epidermis, we used 4′,6-diamidino-2-phenylindole (DAPI) nuclear counterstain (Life Technologies) at 1:500 dilution to image cell nuclei and combined it with SHG imaging to visualize COLI fibres present uniquely in the dermis, which allowed for a clear distinction between the epidermis and dermis. The epidermal thickness was probed as the distance between the top and the bottom of the epidermis in 10 locations 150 µm apart using a line measurement tool in Fiji. ROIs of 250 μm along the epidermis were used to measure the percentage of Ki67-positive proliferating cells in the epidermis compared to the total number of DAPI-stained nuclei. Samples from patient 2 and the STSG patient were excluded from the analysis of proliferating cells due to suspected tissue damage. We used staining for collagen type IV (COLIV) present in the basement membrane to analyze the epidermal-dermal junction (EDJ). Using Fiji, we manually defined the coordinates of the boundary between the epidermis and dermis along the EDJ and imported them into a Jupyter computational notebook. We quantified interdigitation using python code that calculates the arc-chord ratio by dividing the boundary into equal length sections and dividing the arc length along the EDJ by the end-to-end distance for each section. We measured the immunofluorescence signal of COLIV across the EDJ by probing the data along 25 µm trajectories traversing the EDJ. Thickness of the basement membrane and abundancy of COLIV were measured as the width and peak of the COLIV intensity profiles.

To analyze morphology of the scar dermis, we selected ROIs of 300 × 300 (µm) from tile scans of SHG images combined with DAPI nuclear counterstain to analyze dermal cell density defined as the number of cells per mm^2^. Similarly, we selected ROIs of 300 × 300 (µm) from tile scans of immunofluorescence images stained for collagen type IV (COLIV) and used AngioTool software to assess the percentage vessel density^61^. The analysis of vessel density was performed using image batch processing and thus was blinded.

### Transcriptomic analysis

To isolate total RNA, the scar dermis from four timepoints (0, 2, 4, and 6 months) was dissected under a stereo microscope from 20 µm human skin cryosections using 23 G needles. After homogenization with QIAshredder (Qiagen), RNA was isolated using Rneasy Plus Mini Kit (Qiagen) following manufacturer’s instructions. The quality of RNA was tested on an Agilent 2100 Bioanalyzer and samples with the RNA integrity number (RIN) of ≥ 7 were selected. Samples were prepared using Nugen Ovation WTA Pico System V2 and hybridized onto Affymetrix Human Genome U133 Plus 2.0 Array chips in the Bioinformatics UK institute at King’s College London (GEO accession no. GSE145470). The array data were pre-processed using Robust Multiarray analysis (RMA) to perform background correction, normalization, and summarization followed by a baseline transformation (baseline to median of all samples) to generate normalized values. Normalized values were filtered based on their expression, using |fold change| > 1.5 and gene-level *P*-value < 0.01.

To validate the expression changes of selected transcripts in the scar dermis, we isolated total RNA from the third patient involved in the study but not included in the microarray analysis. cDNA was synthesized using OligoDT primers and SuperScript III (Life Technologies). For the quantitative qRT–PCR, PowerUP SYBR Green Master Mix (Life Technologies) was used with primers designed against selected sequences (Supplmentary Table 5). Expression analysis was performed relative to *GAPDH* using the Relative Quantification app (Thermofisher Connect), with gene expression in the scar dermis before transplantation (0 mo) used as a baseline comparison (value = 1).

### In vitro analysis

Reticular fibroblasts were isolated from pieces of discarded scalp skin, obtained from patients after recieving written informed consent. Tissue was washed in Dulbecco’s Modified Eagle’s Medium (DMEM) containing 2% antibiotics–antimycotics (Gibco, Invitrogen) for 20 min, before being transected parallel to the epidermis with a scalpel^62^. The lower/deeper dermis was then minced using scissors, and pieces were left to dry-attach to 35 mm dishes. Media (20% foetal bovine serum (FBS) (A4766801, Thermofisher) and 1% antibiotics–antimycotics in DMEM) was added to dishes which were then maintained at 37 °C in a humidified atmosphere of 5% CO2/95% air for 10 days. After this time, the media was changed to 10% FBS, with 1% penicillin-stretomycin in DMEM.

To establish the effect of hair follicles on reticular fibroblasts, 60,000 reticular fibroblasts were seeded per well of a 24-well plate DMEM containing 10% FBS and 1% penicillin–streptomycin (15140122, Thermofisher). After 24 h, fresh media was added to the cells. A transwell with a pore size of 3 μm size was added to the well and 300 μl of media was added to the transwell. Anagen hair follicles (*N* = 2, *n* = 8) were added to each transwell and incubated for 7 days. Media was changed every 2–3 days to keep both cells and hair follicles alive. After 7 days, RNA was isolated from reticular fibroblasts. The RNA was used to generate cDNA, and RT-PCR was performed to analyze expression of TGF-β1 and IL-6.

### In vitro scar model

We cultured reticular fibroblasts in 24-well plates at 60,000 cells per well in a standard DMEM with 1x Glutamax (Thermofisher), 1% penicillin–streptomycin and 10% FBS. After 24 h, we replaced the standard medium with a low-serum (0.4% FBS) medium for further 24 h. To induce the scar model, we cultured reticular fibroblasts in a low-serum medium supplemented with 1 nM of TGF-β1, 1.5% ascorbic acid, 56 mg/mL Ficoll 70, and 38 mg/mL Ficoll 400. Fibroblasts cultured in the low-serum medium served as a control. After 7 days in culture, we used immunofluorescence staining to measure the expression of ECM molecules: collagen type I (COLI), collagen type III (COIII), ɑ-SMA, and fibronectin. Human hair follicles were introduced to the scar model, separated from reticular fibroblasts by a 3 μm pore transwell membrane, with 8 hair follicles per transwell.

### Statistics

Statistical analysis was performed using GraphPad Prism software v8 with *P* < 0.05 considered to be statistically significant. D’Agostino Pearson normality test was first used to assess the distribution of dataset. The variances between datasets were subsequently compared using an F test (for 2 groups), or Bartlett’s test (for more than 2 groups). In the case of normal distribution and equal variances, comparisons between groups were made using parametric tests; un-paired two-tailed Student’s t-test for two groups, a one-way analysis of variance (ANOVA) for STSG data followed by Tukey’s multiple comparisons test, or a two-way ANOVA followed by Tukey’s multiple comparisons test to compare data from scalp scars. In case of non-normal distribution and/or unequal variances, comparisons between groups were made using nonparametric tests; two-tailed Mann–Whitney *U* test for two groups, and Kruskal-Wallis followed by Dunn’s multiple comparisons test for more than two groups. To identify outliers in the datasets, Grubbs’ test with significance level = 0.05 was used. Numbers of patients and technical repeats are specified in each figure legend. *N* = number of patients, while *n* = number of tissue sections/patient analyzed.

### Reporting summary

Further information on research design is available in the Nature Research Reporting Summary linked to this article.

## Supplementary information

Supplementary Material

Reporting Summary

## Data Availability

Transcriptional data can be found on the GEO database—GEO accession number GSE145470. Requests for other data can be made to C.A.H.
