# Supplementary material for: Anagen hair follicles transplanted into mature human scars remodel fibrotic tissue
Source: NPJ Regen Med. 2023 Jan 6;8:1. doi: 10.1038/s41536-022-00270-3 (PMC9822907; doi:10.1038/s41536-022-00270-3)
Supplement: Supplementary file 1 — Supplementary Material [file 41536_2022_270_MOESM1_ESM.pdf]

SUPPLEMENTARY INFORMATION

Supplementary Results

Anagen hair follicles remodel a split-thickness skin graft scar

Among individuals receiving treatment in the same hair transplantation clinic, there was a patient with a normotrophic scar on his thigh formed after a split-thickness skin graft (STSG) was performed 50 years prior, to heal a deep burn wound (Supplementary Figure 1b, Supplementary Table 1). Autologous scalp anagen hair follicles were transplanted into the STSG scar, and a biopsy was taken at 0 (baseline), 2, 4 and 6 months, consistent with the assessment of post-surgical scalp scars (Supplementary Figure 1b). The clinical photographs and images of H&E staining show that the transplanted hair follicles continued to produce fibers, and all follicles were found in anagen throughout the follow-up period (Supplementary Figure 1b and 2).

We first tested the effect of hair follicle transplantation on the epidermis of an STSG scar (Supplementary Figure 4a) and found a similar effect to scalp scars, with a 2.5-fold ($P < 0.0001$) increase in the epidermal thickness of the scar already at 2 months post-transplant (Supplementary Figure 4b). After just 2 months, the EDJ interdigitation and basement membrane thickness increased 2.5 fold ($P < 0.0001$) and 1.2-fold ($P < 0.0001$), respectively (Supplementary Figure 4c-e). Similarly, the peak intensity of COLIV expression was higher at 4 (1.6-fold, $P < 0.0001$) and 6 months (1.4-fold, $P < 0.005$) post-transplant compared to the baseline (0 months) (Supplementary Figure 4f).

Next, to identify changes to the morphology of the dermis of an STSG scar after hair follicle transplantation, we used DAPI nuclear counterstain combined with the SHG imaging to visualize COLI. At 2 months after hair follicle transplantation, we observed a 1.9-fold

increase (P < 0.0005) in dermal cell density (Supplementary Figure 5a, b). In terms of vascularization, an STSG scar after transplantation of anagen hair follicles showed a more dramatic response than scalp scars, with a 2.4-fold increase (P < 0.05) in vascularization (Supplementary Figure 5c, d), compared to an average 1.6-fold increase (P < 0.0001) in scalp scars at 2 months post-transplant (Figure 3d). This effect could be explained by lower baseline vessel density (at 0 months) in an STSG scar as compared to scalp scars (P < 0.001) (Supplementary Figure 6).

We also compared the morphology COLI fibers in the dermis of an STSG scar before and after hair follicle transplantation. Already at 2 months post-transplant, there was a 1.1-fold decrease in the collagen fraction (P < 0.001) (Supplementary Figure 7a) and a 1.1-fold decrease in the proportion of thick fibers (P < 0.001) (Supplementary Figure 7b). While there was no difference in collagen alignment (Supplementary Figure 7c, d) there was a 2.0-fold increase in the SHG intensity, which is used as a proxy measurement for tension in collagen fibers (P < 0.001) (Supplementary Figure 7e, f). Despite some changes by 2 months post-transplant, none of the changes to the architecture of COLI within the scar dermis after hair follicle transplantation remained present at 6 months post-transplant (Supplementary Figure 7). The lack of the long-term effect of hair follicle transplantation on collagen morphology suggests that in contrast to post-surgical scalp scars, collagen remodeling in an STSG scar may be only transient. This observation can be explained by to the considerably bigger size of the STSG scar compared to scalp scars (Supplementary Figure 1b), which can cause the locally remodeled collagen fibers to be influenced by the surrounding, much more extensive, fibrotic extracellular matrix.

Supplementary Figures

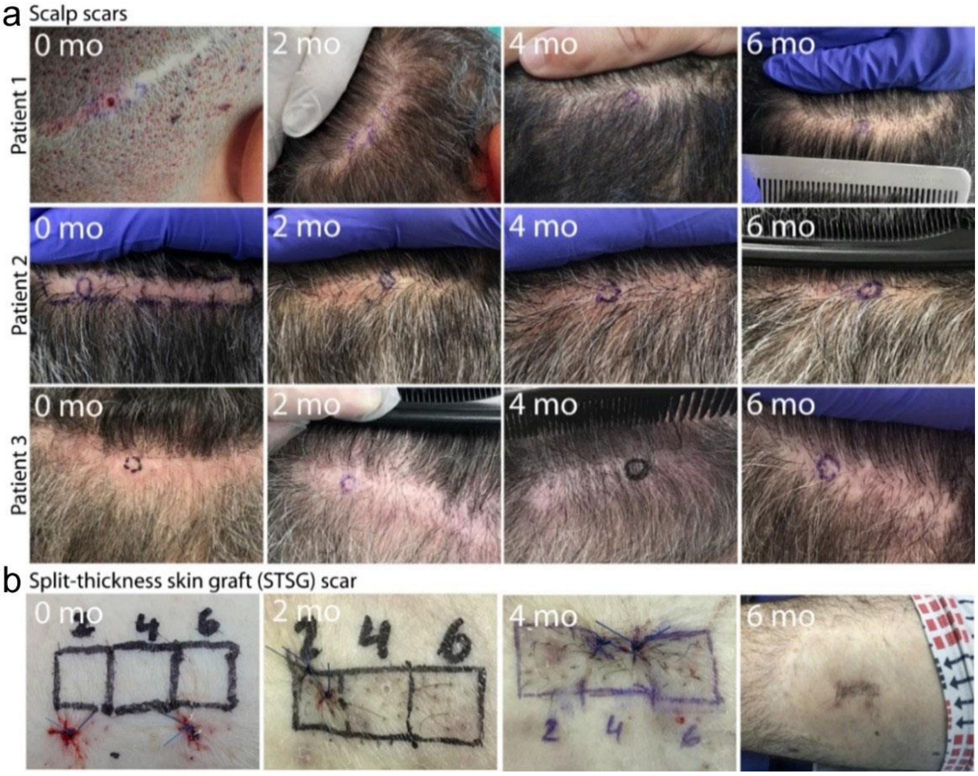

Supplementary Figure 1. Hair follicles transplanted into mature scars continue to grow. (**a**) Three patients undergoing hair follicle transplantation into mature scars on occipital scalps formed postsurgically after a strip of skin was excised from the occipital scalp for harvesting hair follicles during a Follicular Unit Transplantation (FUT) surgery. View before (0 mo) and after 2, 4, and 6 months post-transplant. Circles indicate areas where 3 mm full thickness biopsies were taken at four time points. (**b**) Patient with a hairless scar on the thigh resulted from a split thickness skin graft (STSG) transplanted to heal a burn wound. View before (0 mo) and 2, 4, and 6 months after hair follicle transplantation into scars. Squares indicate areas where the 3 mm full thickness biopsies were taken at 4 different time points.

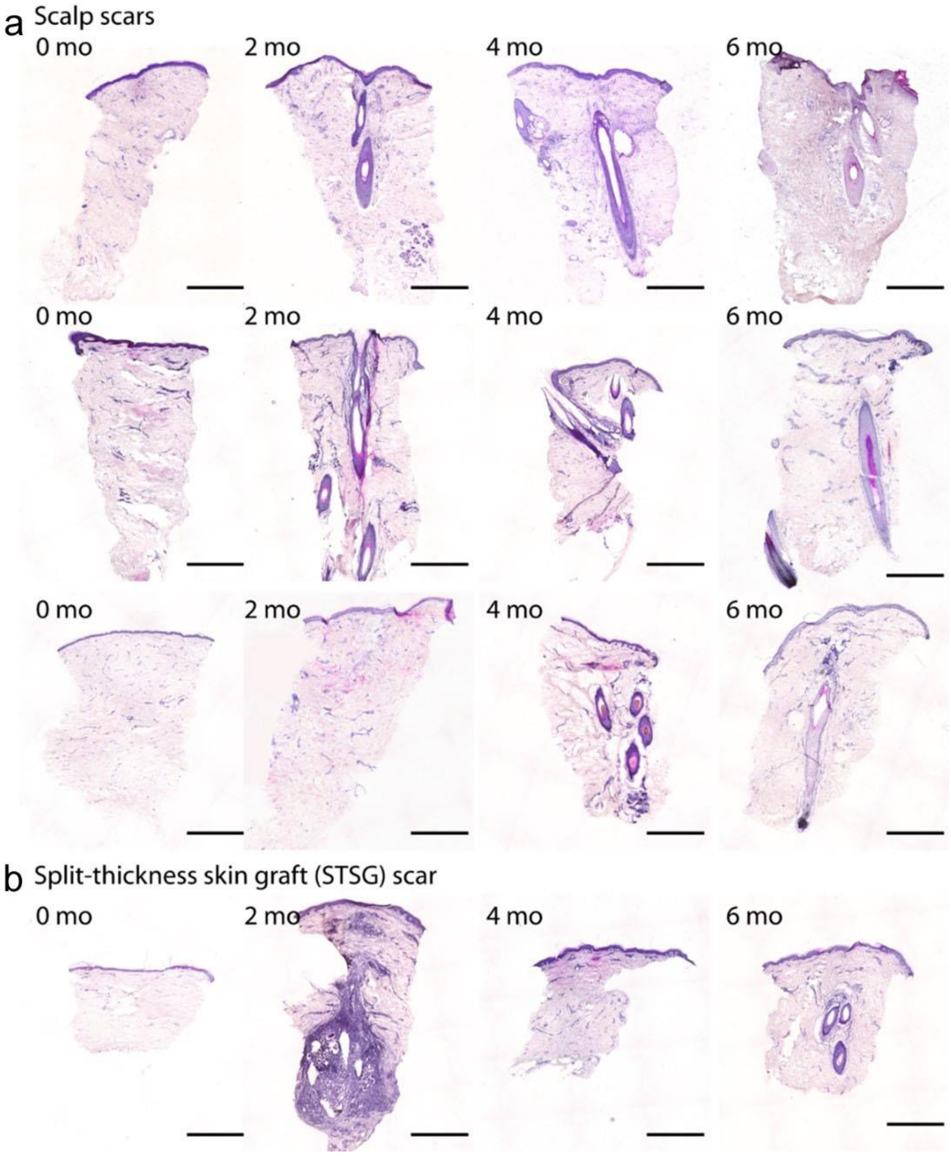

Supplementary Figure 2. Representative haematoxylin and eosin (H&E) images of longitudinal sections of skin biopsies. According to the previous studies, we expected some hair follicles to enter the cycle upon stress-induced transplantation and transition into a new anagen by 60 days post-transplant. Using H&E staining on longitudinal scalp scar (**a**) and STSG (**b**) sections, we found that already by 2 months, all follicles were in anagen. Scale = 1000 µm

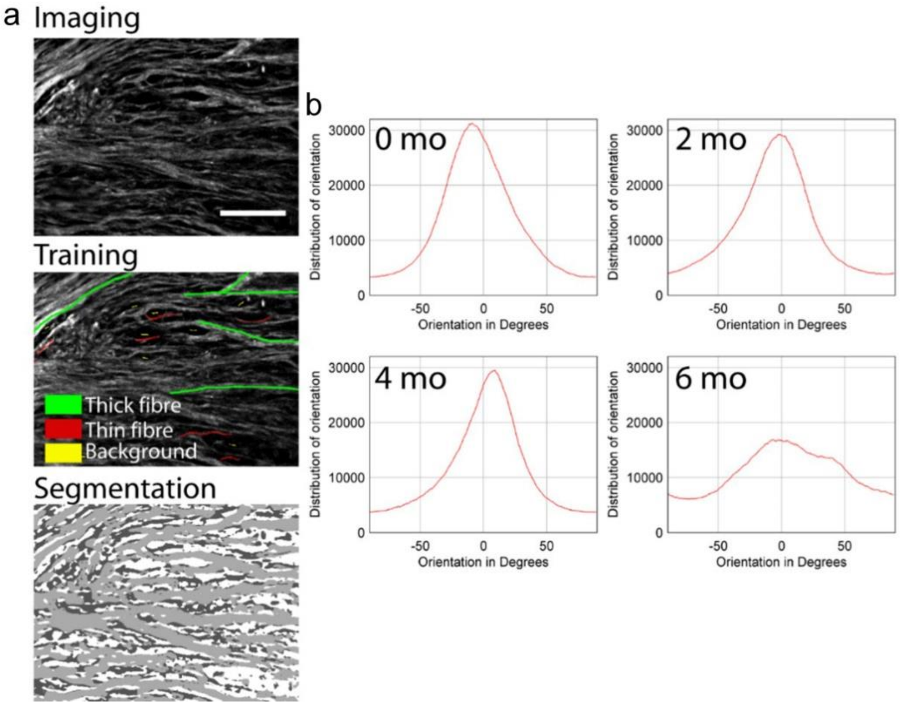

Supplementary Figure 3. Analysis of COLI in the dermis of scars before and after hair follicle transplantation. (**a**) After imaging collagen type I (COLI) fibers using second harmonic generation (SHG) microscopy, we trained the pixel classification software ilastik to recognize morphological features and separate SHG images into thick collagen fibers (defined as width > 10 µm), thin fibers (< 10 µm), and the background. Based on that, the SHG images were then segmented into three channels for further analysis. (**b**) The graphs of orientation angles of COL1 fibers revealed strong peaks around 0° before hair follicle transplantation (0 mo) and up until 4 months after transplantation, suggesting fiber orientation parallel to the epidermis. A higher spread of orientation angles at 6 months post-transplant indicates more disorganized fibers.

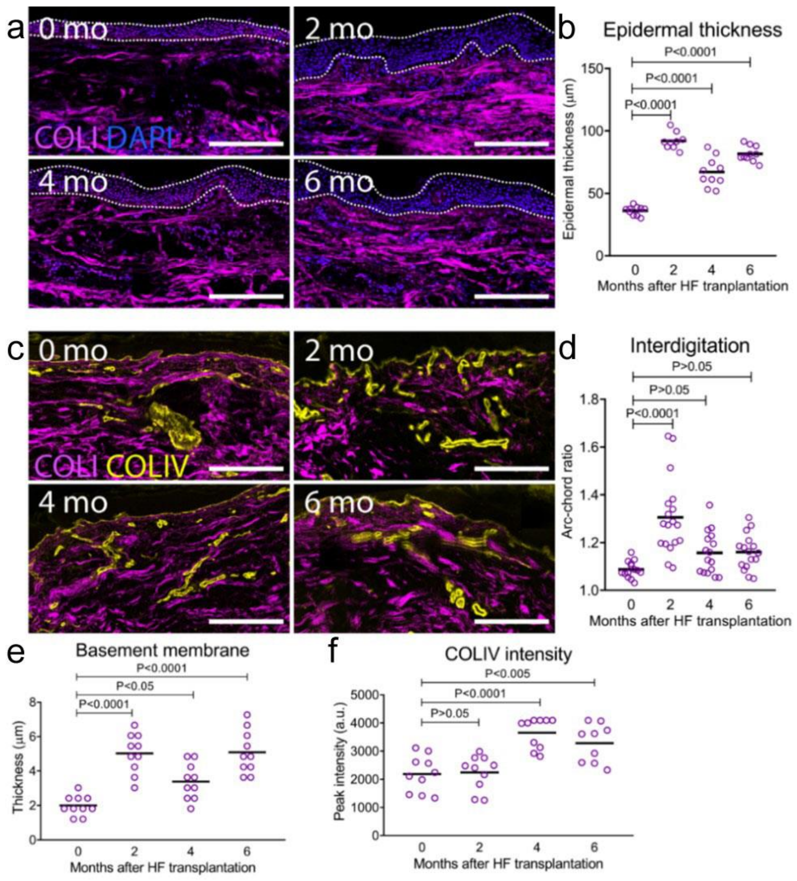

Supplementary Figure 4. Anagen hair follicles remodel the epidermis of an STSG scar. (**a**) Immunofluorescence imaging of 10 μm-thick sections of a mature split thickness skin graft (STSG) scar before (0 mo) and at 2, 4, and 6 months post-transplant of anagen hair follicles. (**b**) We observed an increase in the epidermal thickness is observed at 2, 4, and 6 months after hair follicle (HF) transplantation into the STSG scar. (n = 14, N = 1). (**c**) Epidermal-dermal junction (EDJ) stained for collagen type IV (COLIV) present in the basement membrane. (**d**) The arc-chord ratio revealed increased interdigitation at 4 and 6 months after transplantation of hair follicles (n = 14, N = 1). (**e**) Thickness of the basement membrane was measured as the width of the intensity peak and peak intensities were recorded. The thickness of the basement membrane increases at 2, 4, and 6 months post-transplant as compared to a mature STSG scar (**f**) while the peak intensity was higher after 4 and 6 months (n = 10, N = 1). Reported P values are based on one-way ANOVA tests. Representative images from one patient are shown. Scale bars = 200 μm.

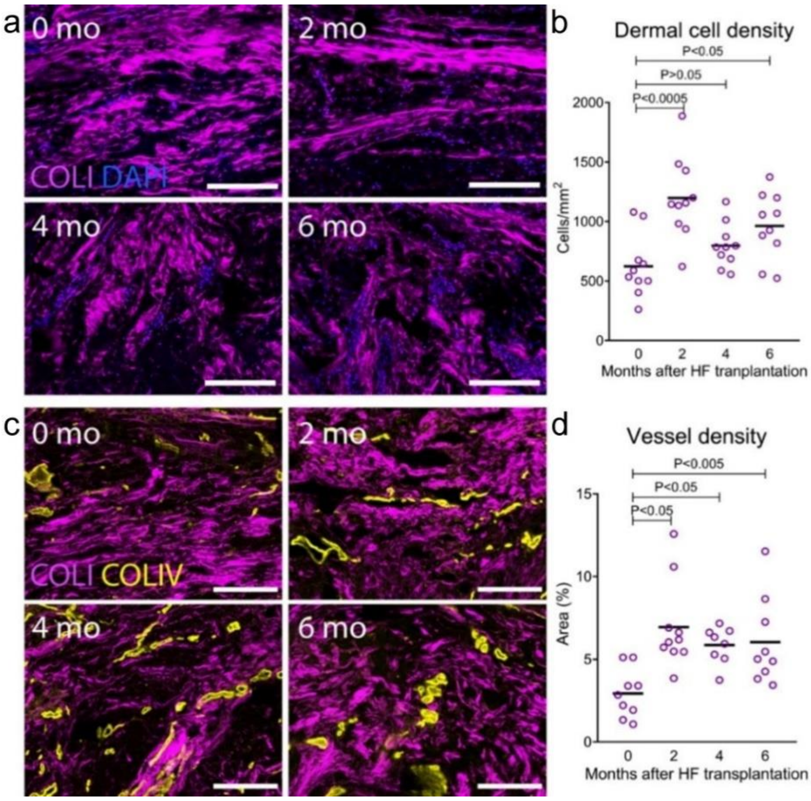

Supplementary Figure 5. Anagen hair follicles remodel the dermis of an STSG scar. (**a**) Immunofluorescence imaging of 10 μm-thick sections of of a split thickness skin graft (STSG) scar before (0 mo) and at 2, 4, and 6 months post-transplant of anagen hair follicles. We used DAPI staining to quantify cell nuclei within the dermis and combined it with second harmonic generation (SHG) imaging to visualize collagen type I (COLI) fibers present uniquely in the dermis. (**b**) Quantification of DAPI stained nuclei in the dermis revealed an increase in the dermal cell density at 2, 4, and 6 months after hair follicle (HF) transplantation (n = 20, N = 1). (**c**) Immunofluorescence imaging of scar sections for collagen type IV (COLIV) present in the basement membrane of blood vessels combined with SHG imaging. (**d**) We quantified the area (%) covered by COLIV staining and found that the dermis of a STSG scar contains higher vessel density after hair follicle transplantation (n = 20, N = 1). Reported P values are based on one-way ANOVA tests. Representative images from one patient are shown. Scale bars = 200 μm.

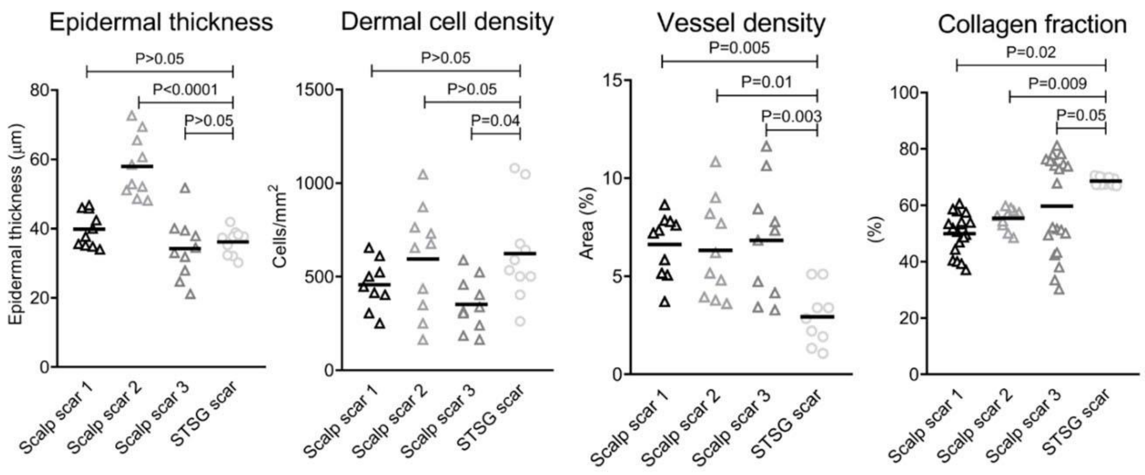

Supplementary Figure 6. Comparison of an STSG scar and scalp scars before hair follicle transplantation. Vessel density (n = 9 measurements) and collagen fraction (n = 10) are lower in the split thickness skin graft (STSG) scar as compared to scalp scars. In contrast, the epidermal thickness (n = 10) and dermal cell density (n = 9) stays the same across scar types. Reported P values are based on one-way ANOVA tests.

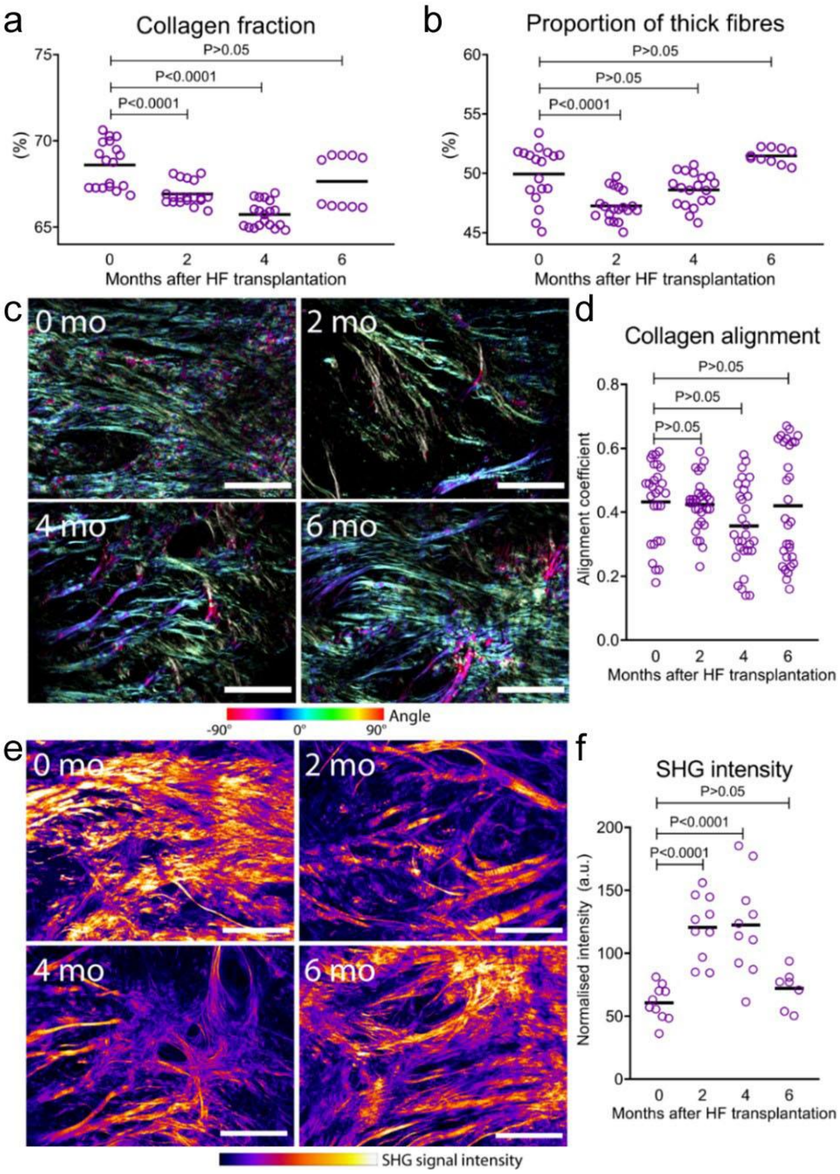

Supplementary Figure 7. Anagen hair follicles remodel the architecture of COLI in an STSG scar. (**a**) Analysis of SHG images reveals an increase in the total fraction of collagen (**b**) and proportion of thick fibers at 2, and 4 months after hair follicle (HF) transplantation into a split thickness skin graft (STSG) scar (n = 36, N = 1). (**c**) Colour-coded maps of fiber orientation show preferential alignment of fibers along the epidermis. (**d**) No difference in collagen organization was found after hair follicle transplantation into a STSG scar. (n = 18, N = 1). (**e**) The SHG signal intensity, used as a proxy measurement for tension in collagen fibers (**f**) decreases at 2 and 4 months after hair follicle transplantation (n = 20, N = 1). Reported P values are based on one-way ANOVA tests. Scale bars = 200 µm.

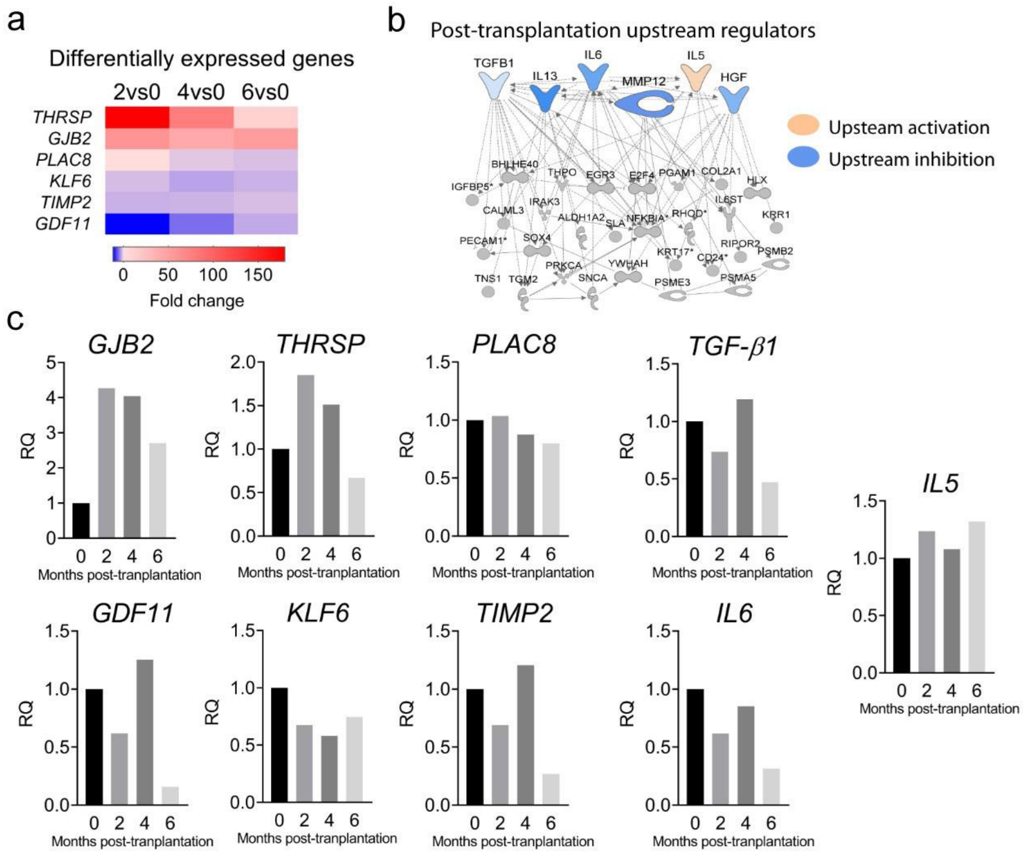

Supplementary Figure 8. **Quantitative RT-PCR validation of microarray results.** (**a**) Candidate genes were selected for validation from the database based on the highest fold change (*GJB2*, *THRSP*, *GDF11*), involvement in cellular functions that are relevant for the study (*TIMP2*, *PLAC8*, *KLF6*), (**b**) and being detected as upstream regulators pre- and post-transplantation using the Ingenuity Pathway Analysis software (*TGF-β1*, *IL5*, *IL6*). (**c**) We used total RNA isolated from the scar dermis of the third patient involved in the study but not included in the microarray analysis to validate the expression changes of selected transcripts. GAPDH was used as a housekeeping control. RQ = relative quantification

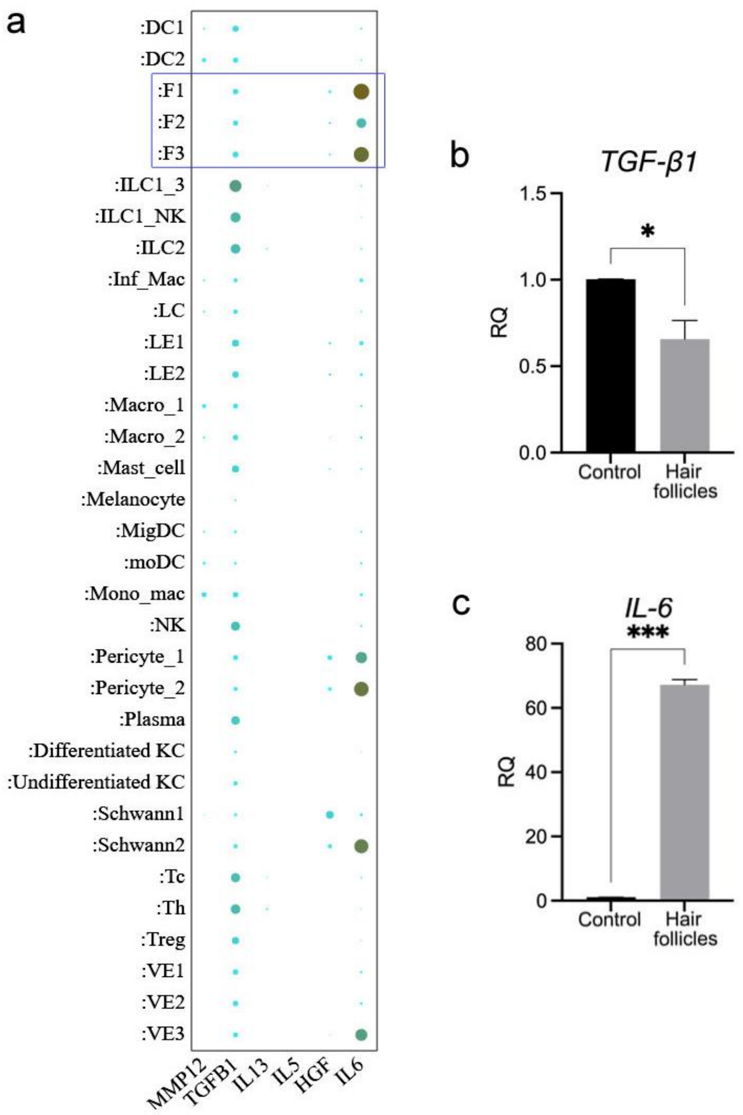

Supplementary Figure 9. Hair follicles reduce the expression of TGF-β1 in fibroblasts. (**a**) Dot plot showing expression of 6 predicted regulators of scar remodelling in human skin. Fibroblast (F) cell types surrounded by blue box. Data taken from [1] and visualised with Adifa (https://app.cellatlas.io/diseased-skin) (b) Expression of TGF-β1 decreases in reticular fibroblasts after 7 days co-culture with anagen hair follicles (N=2). (c) Expression of IL-6 increases in reticular fibroblasts after 7 days co-culture with anagen hair follicles (N=2). Data was analysed using a student's t-test. * $P \leq 0.05$, *** $P \leq 0.001$

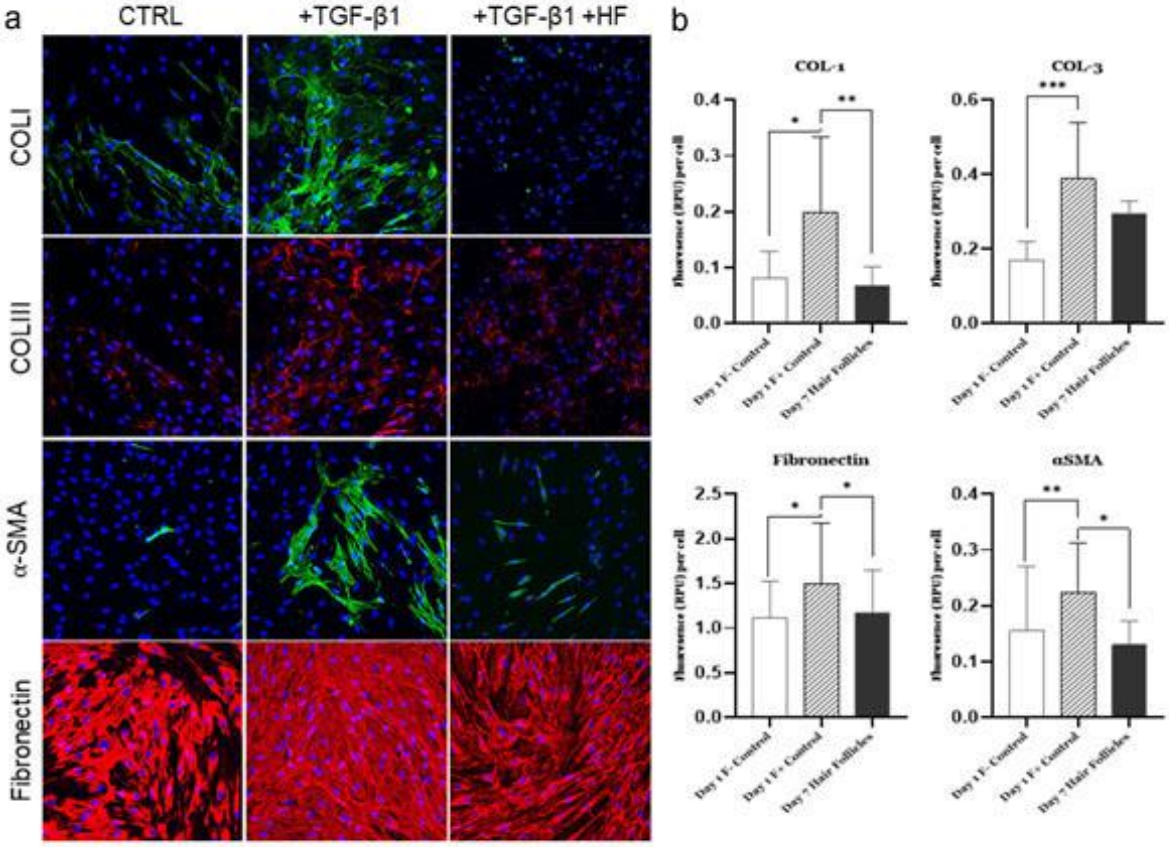

Supplementary Figure 10. Hair follicles reduce the expression of extracellular matrix (ECM) molecules in reticular fibroblasts. (**a**) Representative images of the expression of collagen type I (COLI), collagen type III (COLIII), α-SMA and fibronectin in reticular fibroblasts after 7 days of no treatment (CTRL), treatment with pro-fibrotic TGF-β1, and treatment with TGF-β1 in the presence of human hair follicles (HF). (**b**) We measured the mean immunofluorescence signal of ECM molecules per cell in the population of reticular fibroblasts and found that the presence of HF reduced the expression of ECM molecules. * P ≤ 0.05, ** P ≤ 0.01, *** P ≤ 0.001

Supplementary Tables

Patient	Sex	Scar age	Previous skin treatment	Scar width
Scalp scar 1	M	9 year	None	7 mm
Scalp scar 2	M	4 years	None	7 mm
Scalp scar 3	M	14 years	None	10 mm
STSG scar	M	50 years	Split thickness skin graft	not measured

Supplementary Table 1. Patient information. STSG = split thickness skin graft

Upstream regulator	Molecule type	P-value	Target molecules in dataset
MMP12	peptidase	0.00006	*NFKBIA, PSMA5, PSMB2, PSME3, YWHAH*
TGF-B1	growth factor	0.0004	*BHLHE40, CALML3, COL2A1, IGFBP5, NFKBIA, PECAM1, PRKCA, RHOD, SOX4, TGM2, THPO*
IL13	cytokine	0.006	*ALDH1A2, IRAK3, SLA, SNCA, TGM2, TNS1*
IL5	cytokine	0.009	*CD24, EGR3, RIPOR2*
HGF	growth factor	0.01	*HLX, KRR1, KRT17, PGAM1*
IL6	cytokine	0.03	*E2F4, IL6ST, NFKBIA, TGM2*

Supplementary Table 2. Upstream regulators of the core transcriptional signature of the scar dermis after hair follicle transplantation.

Antibody	Source	Catalog number	Host Species	Dilution
Anti-Collagen type IV (COLIV)	Abcam	ab6311	Mouse	1:200
Anti-Ki67	Abcam	ab15580	Rabbit	1:1000
Anti-Collagen type I (COLI)	Abcam	ab6308	Mouse	1:200
Anti-Collagen type III (COLIII)	Abcam	ab7778	Rabbit	1:200
Anti-Fibronectin	Sigma	F3648	Rabbit	1:100
Anti-αSMA	Abcam	ab7817	Mouse	1:50

Supplementary Table 3. List of primary antibodies.

Antibody	Source	Catalog number	Host Species	Dilution
Anti-rabbit Alexa Fluor 546	Life Technologies	A21207	Goat	1:500
Anti-mouse Alexa Fluor 546	Life Technologies	A21203	Goat	1:500
Anti-mouse Alexa 488	Thermofisher	Z25002	Goat	1:500
Anti-rabbit Alexa 568	Thermofisher	Z25306	Goat	1:500

Supplementary Table 4. List of secondary antibodies.

Gene	Forward primer (5’ to 3’)	Reverse primer (5’ to 3’)
GAPDH	CGTCTTCACCACCATGGAGA	CGGCCATCACGCCACAGTTT
GJB2	AAAGCCAGGTTCCACAGAGG	ACAACAGACAGCCTCTCAGC
THRSP	AGCGTTACCCCAAGAACTGC	TGCGGTTCCATTCTCTTCGC
PLAC8	GAACAAGCGTCGCAATGAGG	AACCCACATGTTCTGAGAGGC
GDF11	TTCCCGCGAACATCACACC	AGTGGAAGAAAGGAGGCTTGG
KLF6	GGCAGCGGAGCTTTGAATAGG	AGGCTGAAACATAGCAGGGC
TIMP2	CAGCTTTGCTTTATCCGGGC	TGTCACCAAAGCCACCTACC
TGFB1	TACCTGAACCCGTGTTGCTCTC	GTTGCTGAGGTATCGCCAGGAA
IL6	CTTCGGTCCAGTTGCCTTCTCC	ATGCCGTCGAGGATGTACC
IL5	CCAAAGGCAAACGCAGAACG	GTTTGACTCTCCAGTGTGCC

Supplementary Table 5. List of RT- PCR primers.

Supplementary References

1. Reynolds, G., *et al.* Developmental cell programs are co-opted in inflammatory skin disease. *Science* **371**(2021).
